# Overexpression of wild type or a Q311E mutant *MB21D2* promotes a pro‐oncogenic phenotype in HNSCC

**DOI:** 10.1002/1878-0261.12806

**Published:** 2020-10-15

**Authors:** Daniel E. Gracilla, Praveen Kumar Korla, Ming‐Tsung Lai, An‐Jen Chiang, Wen‐Shiung Liou, Jim Jinn‐Chyuan Sheu

**Affiliations:** ^1^ Institute of Biomedical Sciences National Sun Yat‐sen University Kaohsiung Taiwan; ^2^ Department of Pathology Taichung Hospital Ministry of Health and Welfare Taichung Taiwan; ^3^ Department of Obstetrics and Gynecology Kaohsiung Veterans General Hospital Taiwan; ^4^ Department of Health and Nutrition Biotechnology Asia University Taichung Taiwan; ^5^ School of Chinese Medicine China Medical University Taichung Taiwan; ^6^ Department of Biotechnology Kaohsiung Medical University Taiwan

**Keywords:** cadherin‐binding gene, MB21D2, overexpression, Q311E recurrent mutation, squamous cell carcinoma, cadherin‐binding gene, MB21D2, overexpression, recurrent Q311E mutation, squamous cell carcinoma, cadherin‐binding gene, MB21D2, overexpression, Q311E recurrent mutation, squamous cell carcinoma, cadherin‐binding gene, MB21D2, overexpression, recurrent Q311E mutation, squamous cell carcinoma

## Abstract

Cadherin‐mediated cell–cell contacts regulated by intracellular binders play critical roles in tissue homeostasis and tumorigenesis. Here, we screened mutational profiles of 312 annotated genes involved in cadherin binding in human squamous cell carcinomas and found MB21D2 to carry a unique recurrent Q311E mutation. MB21D2 overexpression was also frequently found in head and neck cancer (HNSCC) and was associated with poor clinical outcomes. Cell‐based characterizations revealed pro‐oncogenic roles for MB21D2 wild‐type (WT) and its Q311E mutant (Q311E) in cell proliferation, colony formation, sphere growth, and migration/invasion by promoting epithelial–mesenchymal transition. Conversely, MB21D2 knockdown in MB21D2‐overexpressing cells resulted in cell growth arrest and apoptosis. Xenograft tumor models with Q311E‐expressing cells formed larger and more aggressive lesions, compared to models with WT‐MB21D2‐expressing cells or an empty vector. Transcriptome and protein interactome analyses revealed enrichment of KRAS signaling by MB21D2 expression. Immunoblotting confirmed RAS elevation, along with upregulation/phosphorylation of PI3K, AKT, and CREB. Blocking RAS signaling in MB21D2‐expressing cells by manumycin significantly reduced cell growth and survival. Our study thus defined RAS signaling‐dependent pro‐oncogenic roles for MB21D2 overexpression and Q311E MB21D2 expression in HNSCC development.

## Graphical abstract

A novel cancer gene MB21D2, a known intracellular cadherin binder, was found to harbor Q311E recurrent mutation and to be overexpressed in head and neck cancer (HNSCC). Wild‐type MB21D2 and its mutant form mediate pro‐oncogenic activities and promote EMT. Blockage of RAS could be used as a strategy for treating cancer with MB21D2 overexpression or mutation, particularly for tumors resistant to DNA‐damaging drugs.

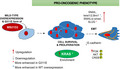

**Abbreviations**
GEPIA: gene expression profiling interactive analysis
HNSCC: head and neck squamous cell carcinoma
KRAS: Kirsten rat sarcoma viral oncogene homolog (KRAS proto‐oncogene, GTPase)
MB21D2: Mab‐21‐containing domain 2
PI3K: phospho‐inositol kinase 3
Q311E: change in glutamine at the position 311 to glutamic acid
SCCs: squamous cell carcinoma(s
TCGA: The Cancer Genome Atlas
WT: in this study, wild‐type MB21D2

## 1 Introduction

Cadherin‐mediated cell adhesion and migration play vital roles in controlling epithelial cell behaviors, and aberrations in the components of cadherin complex have been implicated in cancer development and invasion [1, 2]. For instance, genetic loss or epigenetic silencing of E‐cadherin (CDH1), which has been shown as a tumor suppressor, was frequently detected in cancers [2, 3, 4]. In contrast, certain cadherins, such as R‐cadherin (CDH4) and K‐cadherin (CDH6), can activate oncogenic pathways critical to cancer progression by regulating the activity of downstream effectors [5, 6]. Recent studies indicated the emerging roles of intracellular cadherin binders in the establishment of cadherin‐mediated pro‐ or anti‐oncogenic signaling networks [7, 8]. In particular, some cadherin binders can transduce downstream signaling by controlling the activity of several key intracellular kinases, such as RACK1‐PKC and/or RACK1‐MAPK axes [9]. Some can even translocate into the cell nucleus and complex with different transcription factors to regulate gene expression, for example, β‐catenin [10]. MACF1, a well‐known cadherin binder capable of controlling the stabilization of AXIN/β‐catenin complex, regulates β‐catenin release and translocation into the nucleus [11]. These findings indicate that cadherin binders serve as direct or indirect regulators of gene expression in cells and play potent roles in tumorigenesis.

Recurrent mutation is an important genetic feature of a known oncogene resulting from selective pressure upon dysregulation in cellular functions [12, 13]. Because recurrent/hotspot mutations usually locate at functional domains of an oncogene, such substitutions suggest a mechanism for oncogene activation that mimics oncogene overexpression during cancer development. Such activating mutations can contribute to clonal selection or expansion during cancer cell evolution, leading to oncogene addiction [14, 15]. The typical example would be *PIK3CA* recurrent mutations, which constitutively activate the catalytic subunit (p110) of PI3K [12, 16, 17], and in turn promotes dysregulated cell proliferation, uncontrolled motility, and evading apoptosis. Several signaling molecules involved in oncogenesis, for example, RAS, RAF, and AKT, were also found to harbor recurrent/hotspot mutations at critical sites of the sequences in cancer lesions [18, 19]. Due to functional relevance of those unique mutations, the 20/20 rule, which means that more than 20% of the mutation events in one gene contribute to silent mutations or a hotspot feature, is widely accepted as the common criterion to distinguish driver mutations from passenger mutations in cancer genomic study [12, 18]. With the achievements of The Cancer Genome Project, newly defined cancer‐associated genes, especially those encoding phosphoproteins, were recently discovered and await further functional characterization [18, 19].

MB21D2 (a.k.a. C3orf59), a Mab21 domain‐containing protein, belongs to a unique protein family involved in a variety of important cellular processes, including cell survival, proliferation, and migration. Studies in simple organisms such as *C. elegans* to higher organisms such as zebrafish, xenopus, or mouse revealed that proteins in this family function as cell‐fate‐determining factors that control organogenesis and embryonic development [20, 21, 22, 23, 24, 25]. In humans, twelve annotated genes have been identified to be capable of forming a compact interactome together even though they may have distinct molecular functions. For example, cGAS (a.k.a. MB21D1), MAB21L1, and MAB21L2 were defined as nucleotidyltransferase enzymes. cGAS is popularly known to (a) act as cytosolic sensors for free nucleic acid or micronuclei during bacterial/viral infections and (b) trigger immune response by activating cGAS‐STING signaling [26, 27, 28, 29]. ITPRIP (a.k.a. DANGER), ITPRIPL1, and ITPRIPL2 were found as key regulators of IP3 signaling by controlling IP3R‐mediated Ca^2+^ release from ER membrane [30, 31]. Furthermore, TMEM102, MIEF1, and MIEF2 participate in mitochondrial organization and regulation in fission/fusion balance; thus, they can determine cell viability [32, 33, 34, 35]. In particular, MB21D2, which was found at the center of the interactome formed by Mab21‐containing proteins, makes connections with members in those different cellular processes, suggesting that MB21D2 functions as a signaling hub in regulating stress‐responsive pathways.

Using quantitative proteomics, MB21D2 was recently found as a novel intracellular binder for E‐cadherin [36]. Although the molecular function remains poorly understood, data from the PhosphoSitePlus database (http://www.phosphosite.org/) indicate the involvement of MB21D2 in cellular signaling regulation with several potent phosphorylation sites identified, including one at Y300 next to the Q311 [37]. This finding suggests possible alterations in cellular signaling caused by the Q311E (neutral to negative charge) recurrent mutation which could be detected across squamous cell carcinomas. In addition, advanced machine‐learning‐based studies suggest Q311E substitution in MB21D2 as a potent driver mutation in cancer worthy of a more detailed investigation [18, 38]. In this study, we investigated the oncogenic roles of a novel gene named MB21D2 which harbors a recurrent Q311E mutation which is overexpressed in head and neck squamous cell carcinoma (HNSCC).

## 2 Results

### 2.1 MB21D2 is frequently overexpressed and mutated among cadherin binders found in head and neck squamous cell carcinoma

**Fig. 1:**
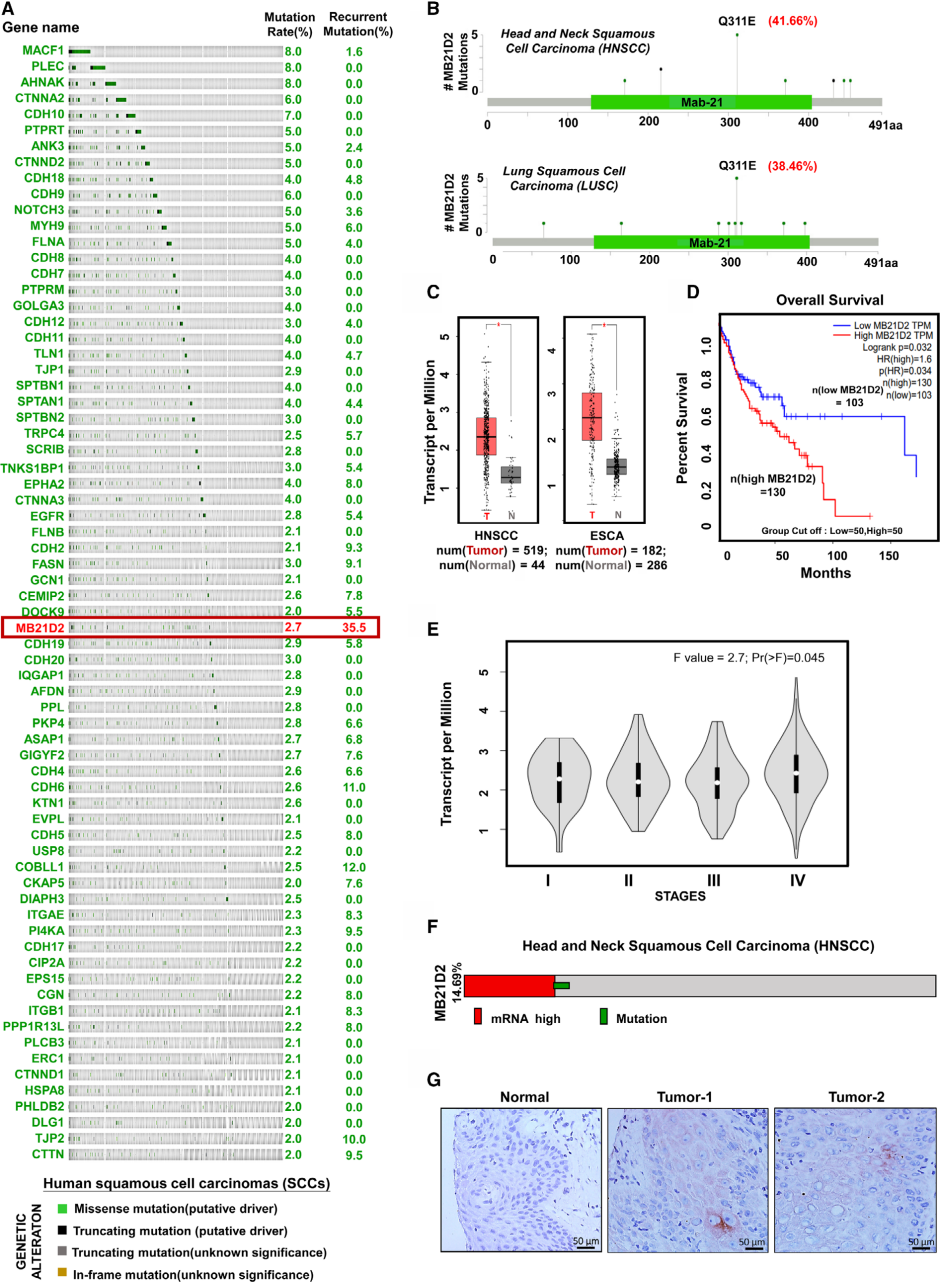
*MB21D2* contains a recurrent Q311E mutation in human squamous cell carcinomas and the impacts of its upregulation on cancer development. (A) Mutation (shown in OncoPrint) and recurrent mutation (number of highest recurrent base substitution over total mutation events within the gene) rates of cadherin‐binding genes filtered at 2% alteration frequency from 1529 patients in four squamous cell carcinomas, namely cervical squamous cell carcinoma (CSCC), esophageal squamous cell carcinoma (ESCA), lung squamous cell carcinoma (LUSC), and head and neck squamous cell carcinoma (HNSCC) collected from TCGA sequencing databank. (B) Mutation profiles indicating hotspot mutation in *MB21D2* and its recurrent mutation (Q311E) in HNSCC, LUSC, and CESC based on sequencing data from TCGA. (C). Comparison of MB21D2 expression at the mRNA levels (log2, TPM + 1) in HNSCC (tumor *n* = 519 with ave. mRNA level of 4.11 and normal *n* = 44 with ave. mRNA level of 1.4) and ESCA [normal *n* = 182 with ave. mRNA level of 3.77 and tumor (*n* = 286) with ave. mRNA level of 1.76] with the normal counterpart. Plotted as mean and SEM (standard error of the mean) using *t*‐test for comparison. (D) Comparison of overall survival times between HNSCC patients with high MB21D2 and patients with low MB21D2. Log‐rank test method was used to estimate survival curves from total patients of *n* = 233 where high MB21D2 is *n* = 130 and low MB21D2 is n = 103. (E) Expression plots indicating MB21D2 mRNA levels in cancer lesions at different tumor stages (mean mRNA level in stages I = 2.25, II = 2.20, III = 2.18, and IV = 2.54 expressed in log2, TPM + 1). One‐way ANOVA was carried out to determine mean differential expression in four stage‐wise violin plotting (with SEM) with data of HNSCC patients (n = 238). (F) Heat map showing the percentage of patients carrying high mRNA levels (*n* = 66 accounting for 12.9%) and mutation (*n* = 12 accounting for 2.4 %) in MB21D2 based on TCGA sequencing data (*n* = 510 samples). (G) IHC staining (scaled bar 50 μm) showing protein expression levels of MB21D2 in normal and HNSCC tissues (*n* = 90). The mRNA data in C, D, and E were extracted from the GEPIA database.

To screen for mutational profiles of cadherin‐binding genes, we selected 312 (Table S1) genes, which are annotated as known or putative cadherin binders from the UniProt database (https://www.uniprot.org/). Seventy genes with mutation rates ≥ 2% in four human squamous cell carcinomas (SCCs) were filtered out by using DNA sequencing data from the TCGA database (https://www.cbioportal.org) (Fig. 1A and Table S2). Among them, *MB21D2* exhibited the highest recurrent mutation rate from glutamine (Q) to glutamic acid (E) at the 311 a.a. position, accounting for 35.50% of total mutation events in *MB21D2* across four SCCs (Fig. 1A). Notably, *MB21D2* is the only cadherin‐binding gene with a recurrent mutation rate larger than 20% detected in two SCCs, including head and neck squamous cell carcinoma (HNSCC: 41.66%) and lung squamous cell carcinoma (LUSC: 38.46%) (Fig. 1B). To determine the biological relevance of this mutation, we next analyzed MB21D2 mRNA levels using data from the GEPIA database (http://gepia.cancer‐pku.cn/). We found this gene to be upregulated in the majority of human cancer types (32/33), including all human SCCs (Fig. S1A). In particular, MB21D2 levels in HNSCC and ESCA (esophageal squamous cell carcinoma) were significantly higher than in normal tissues (Fig. 1C). For patients with HNSCC, MB21D2 overexpression was correlated with shorter overall survival times (Fig. 1D) and advanced cancer stages during the progression (Fig. 1E). Based on the TCGA dataset of HNSCC (496 cases), high MB21D2 mRNA levels were found in 12.54% and somatic mutations in 2.15% of the cases, accounting for a total of 14.69 % of HNSCC with genetic alterations in *MB21D2* (Fig. 1F). Upregulation of MB21D2 and Q311E mutation was found in 3 subtypes of HNSCC, which include cancers of oral cavity (high mRNA: 8.8%, Q311E: 1.1%, *n* = 200), oropharynx (high mRNA: 9.1%, Q311E: 6.1%, *n* = 39), and larynx (high mRNA: 22.8%, Q311E: 1.4%, *n* = 89) (Fig. S1B). The average expression level of MB21D2 in tumors with Q311E mutation was found to be similar to that in tumors without mutation (Fig S1C).

As HPV is a major risk factor for HNSCC, we also checked for MB21D2 expression based on HPV infection statuses (the existence of E6/E7 viral markers) and found lower MB21D2 levels in patients with HPV infection as compared with patients without (Fig. S2A, *P* = 0.0471). Then, we compared the expression levels between MB21D2 and p16 (CDKN2A, a known reliable host marker for HPV infection) [39] and found a negative correlation between these two genes (Fig. S2B, *P* = 0.0159). Furthermore, patients with MB21D2 overexpression showed lower p16 levels (Fig. S2C, *P* = 0.0297). These data suggest a negative correlation between *MB21D2* alterations and HPV infection in HNSCC. Specific IHC staining on tissue microarray revealed positive immunostaining of MB21D2 in HNSCC samples but negative in normal epithelia (Fig. 1G). These data suggest the involvement of *MB21D2* overexpression and recurrent Q311E mutation in the development of human SCCs, particularly in HNSCC.

### 2.2 Functional impacts of MB21D2 overexpression and its Q311E mutant on cell proliferation, colony formation, and sphere‐forming activity *in vitro*

**Fig. 2:**
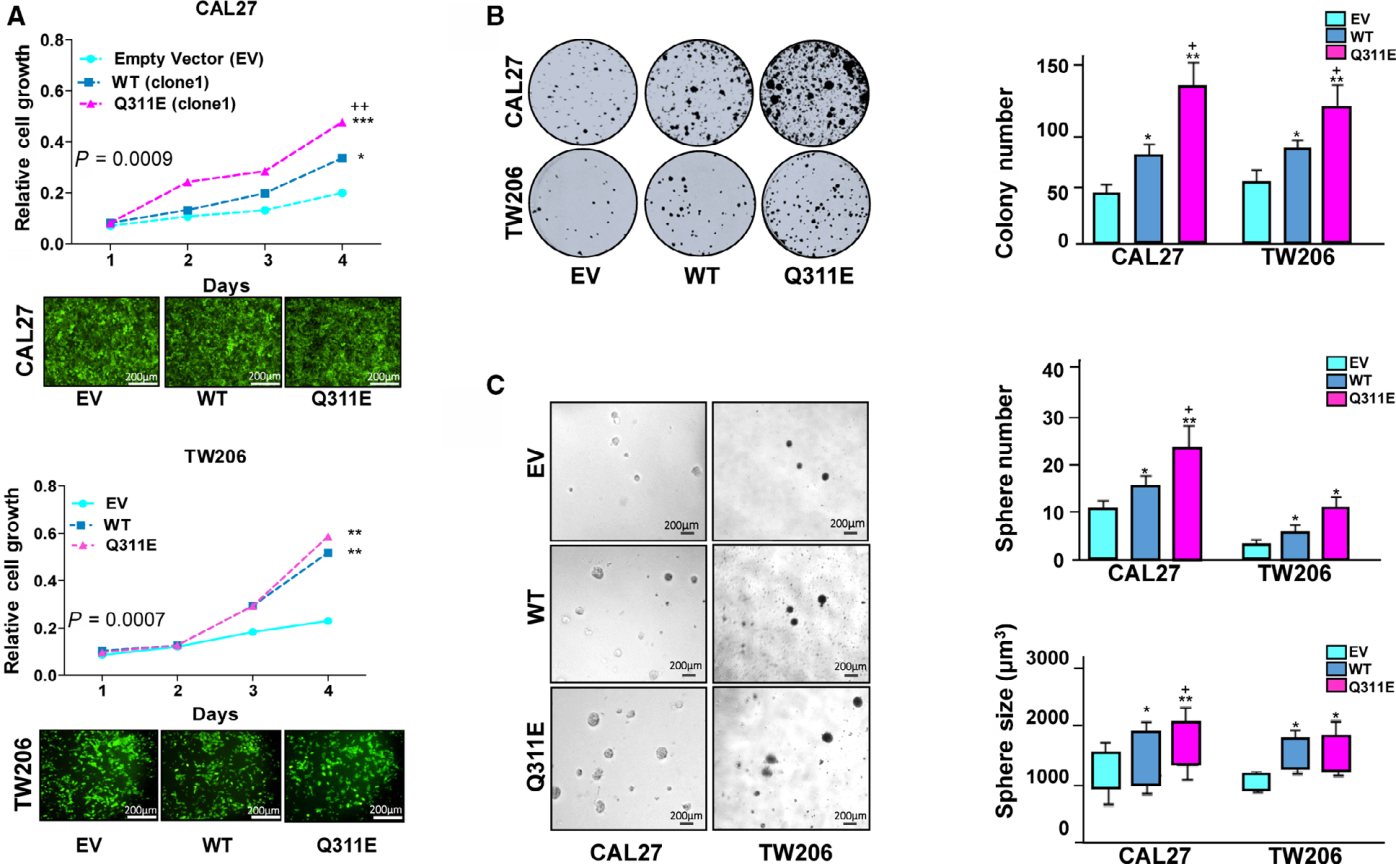
Overexpression of MB21D2 and its Q311E mutant induce cell proliferation, colony formation, and sphere growth in 3D culture. Stable cells in CAL27 and TW206 lines, expressing WT and Q311E MB21D2, were prepared for cell‐based functional studies. Cells carrying the empty vector were utilized as control (EV). (A) Cell proliferation was monitored using CAL27 (upper panel) and TW206 (lower panel) cell clones (scaled bar 200 μm) for four days by MTT assay. Two‐way ANOVA validated by Tukey’s test of significance was carried out to determine significant difference on proliferation and mean comparison among groups. Data are presented as averages ± SD of 6 replicates. (B) CAL27 and TW206 cells were seeded in 30‐mm dishes at low cell density (500 cells/dish) and cultured at 37 °C for 7 days. After cell fixation and staining with crystal violet, representative images (left‐hand panel) were taken, and colony number was counted. The plot indicates the average colony numbers ± SD from three independent experiments (right‐hand panel). (C) Cells expressing different constructs were cultured in soft agar for 14 days. Representative images (scaled bar 200 μm) for sphere growth were taken (left‐hand panel), and the numbers and sizes of the spheres were measured. For Figure B and C, one‐tailed t‐test was used to determine the statistical significance between groups. The plots indicate the average sphere numbers (right upper panel) and sizes (right lower panel) ± SD from three independent experiments. Statistical significance (EV control vs WT or Q311E): *P < 0.05; **P < 0.01; ***P < 0.001. (WT vs Q311E): ^+^ *P* < 0.05; ^++^ *P* < 0.01; ^+++^ *P* < 0.001.

To determine the possible impact of *MB21D2* overexpression and recurrent mutation on cellular behaviors, we performed transient expression study in HNSCC cells followed by validation with stable cell clones that constitutively express wild‐type (WT) and the mutant (Q311E) MB21D2 (Fig. S3). CAL27 and TW206 cells were selected as cell line models due to their relatively low MB21D2 levels among all cell lines tested (Fig. S4). In transient expression experiments, cells with Q311E expression showed higher cell proliferation rate and formed bigger colonies as compared with cells with an empty vector (upper and lower left panels in Fig. S5A and Fig. S5B). Interestingly, WT MB21D2 overexpression, in a reverse way, slowed down cell growth and suppressed colony‐forming activity in transfected cells (upper and lower left panels in Fig. S5A and Fig. S5B). However, in stable CAL27 and TW206 cell clones, both WT‐ and Q311E‐expressing cells exhibited increased proliferation rate than control cells (Fig. 2A). Cells with constitutive WT and Q311E expression exhibited the ability to form more colonies (Fig. 2B) and spheres with bigger sizes in soft agar (Fig. 2C). Similar observation was also found in clone 2 of CAL27 cell lines (Fig. S6A upper and lower panels). Our data indicate that the Q311E substitution may provide more survival advantages as compared to WT, leading to more aggressive phenotypes in HNSCC cells. On the other hand, the results of our transient expression study support a point view that MB21D2‐WT overexpression may serve as a selection barrier to enrich cell clones with tolerance to MB21D2‐induced cell growth arrest/senescence.

### 2.3 MB21D2 knockdown triggered cell growth arrest and apoptosis

**Fig. 3:**
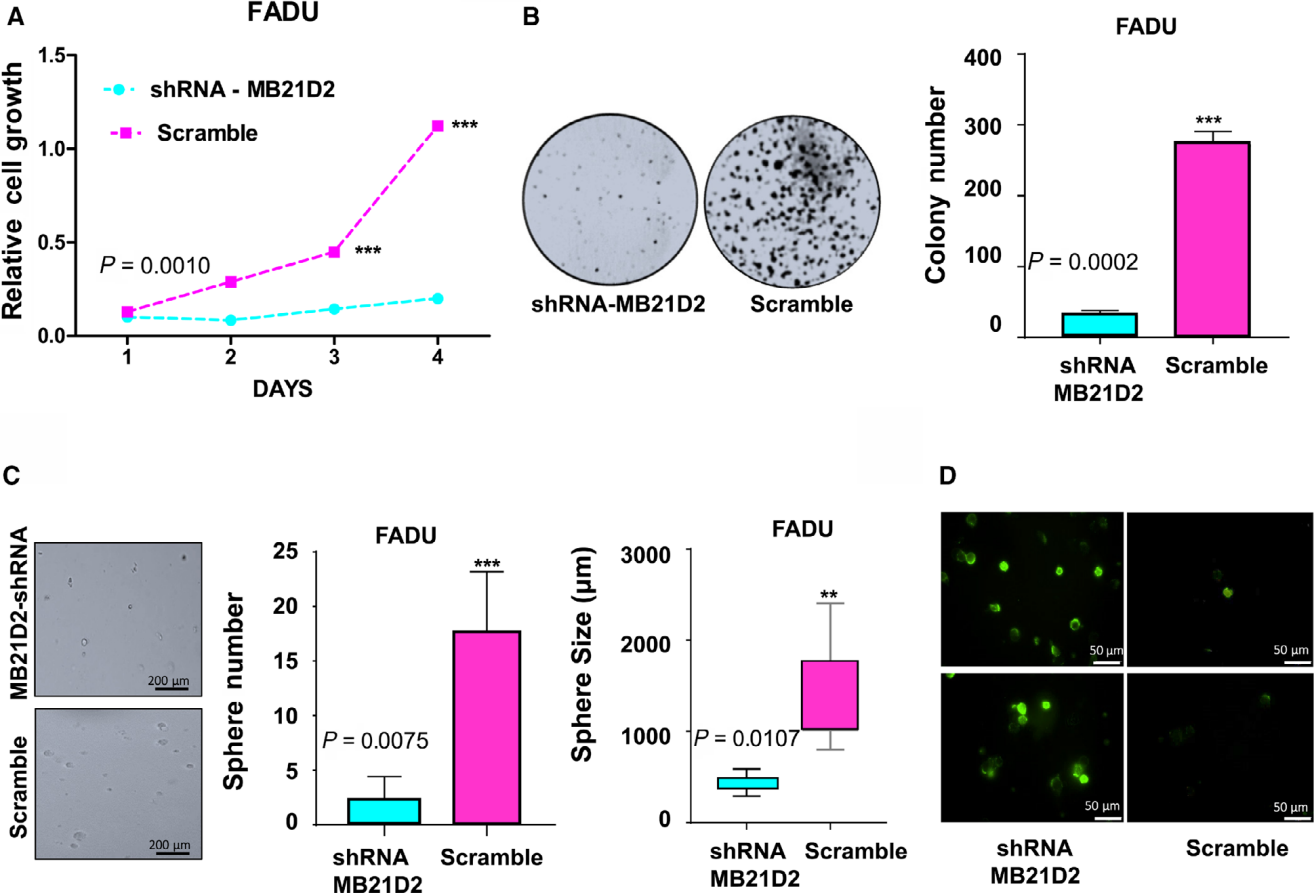
MB21D2 knockdown reduced cell proliferation, colony formation, and sphere growth in FADU (high MB21D2‐expressing) cells. (A) Cell proliferation was monitored for four days on FADU cells transfected with shRNA against MB21D2 by MTT assay. Cells treated with scramble were utilized as control. Two‐way ANOVA validated by Tukey’s test of mean difference was carried out to determine significant differences in proliferation among groups. Data are presented as averages ± SD of six replicates. (B) Treated FADU cells were seeded in 30‐mm dish (500 cells/dish) and cultured for 7 days. Representative images were taken (left‐hand panel), and colony numbers were counted as averages ± SD from three independent experiments (right‐hand panel). (C) Cells transfected with shRNA and scramble constructs were cultured in soft agar for 14 days. The representative images (scaled bar 200 μm) for sphere growth were taken (left‐hand panel), and the numbers and sizes of the spheres were measured. The plots indicate the average sphere numbers (center) and sizes (right‐hand panel) ± SD from three independent experiments. (D) Annexin V staining was performed to detect apoptotic cells (scaled bar 50 μm) in FADU cells 24 h after transfection in collagen‐coated glass‐bottom dish (*n* = 3). For Figure B and C, one‐tailed *t*‐test was used to determine the statistical significance between groups. Statistical significance (EV control vs WT or Q311E): *P < 0.05; **P < 0.01; ***P < 0.001. (WT vs Q311E): ^+^ *P* < 0.05; **^++^** *P* < 0.01; **^+++^** *P* < 0.001.

Since the recurrent Q311E mutation showed the genetic feature of an oncogene and MB21D2 expression promoted clonal selection, we considered the possibility of MB21D2‐induced addiction in cancer cells with MB21D2 overexpression. To prove our concept, FADU cells, showing the highest MB21D2 level among HNSCC cell lines screened, were utilized for gene knockdown study by specific anti‐MB21D2 shRNA. Our data revealed that MB21D2 downregulation suppressed cell proliferation (Fig. 3A), and attenuated colony formation (Fig. 3B) and sphere growth in soft agar (Fig. 3C). Annexin V staining further indicated increased cell death/apoptosis in cells transfected with anti‐MB21D2 shRNAs as compared to control cells treated with scrambled shRNA (Fig. 3D). The same knockdown treatments caused limited suppressing effects on CAL27 and TW206 cells (Fig. S7A and S7B). These results support our hypothesis that MB21D2 overexpression serves as a selective force to select and enrich cell clones with additional survival advantages, and the blockage of MB21D2 activity could be utilized as a strategy for treating cancer cells with *MB21D2* overexpression.

### 2.4 MB21D2 and its Q311E mutant accelerated cell migration and invasion via epithelial–mesenchymal transition

**Fig. 4:**
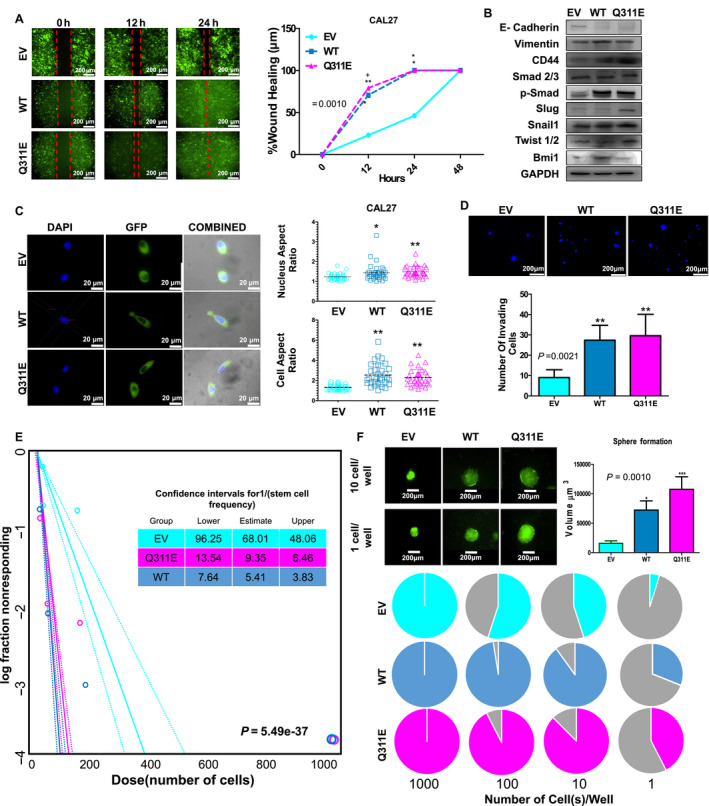
MB21D2 and its Q311E mutant hasten wound healing, increase migration/invasion, possess cancer stemness phenotype, and mediate cell shape change and upregulation of selected EMT/cancer stemness markers. CAL27 line stable cells, expressing WT and Q311E MB21D2, were prepared for wound healing and migration/invasion experiments. (A) Wound healing of CAL27 stable cell lines (1.0 × 10^5^ cells/chamber) monitored every 12 h under 10× magnification (green fluorescence filter) for four days. Representative images (scaled bar 200 μm) were taken (left‐hand panel), and the wound‐healing rate was measured (right‐hand panel). Two‐way ANOVA validated by Tukey’s test of mean difference was carried out to determine significant difference in proliferation among groups. Data are presented as averages ± SD from three replicates. (B) Immunoblot of EMT markers from CAL27 cell lines expressing different constructs. (C) CAL27 stable cell lines (approximately 100 cells) were seeded in a collagen‐coated glass‐bottom dish. 30 single cells (*n* = 30) were subjected to cell and nucleus aspect ratio analysis. Representative images (scaled bar 20 μm) were taken (left‐hand panel), and cell aspect ratio (right upper panel) and nucleus aspect ratio (right lower panel) were measured. (D) Cal27 stable cell lines were allowed to migrate/invade in a 40% matrix gel in a Transwell. Migration/invasion from serum‐free (upper chamber) to 2X serum‐containing media (lower chamber) was measured after 24 h by staining the Transwell filter with DAPI. Representative images (scaled bar 200 μm) were taken under fluorescence microscopy (upper panel), and the number of migrating/invading cells was counted (lower panel). (E) Stemness potential analysis using ‘ELDA: extreme limiting dilution analysis’ using data from anti‐anoikis activity (ultra‐low/nonadherent limiting dilution culture). (F) Sphere image representative (upper left panel) sphere size (upper right panel) and pie chart representative (lower panel) of limiting dilution. Representative image (scaled bar 200μm) of sphere formation (left‐hand panel) and sphere volume (right‐hand panel) were measured after 14 days of culture. For Figure C, D, and F, one‐way ANOVA validated by Tukey’s test was used to determine the statistical significance and differences between groups. Data are presented as averages ± SD from 3 replicates. Statistical significance (EV control vs WT or Q311E): *P < 0.05; **P < 0.01; ***P < 0.001. (WT vs Q311E): +*P* < 0.05; ++*P* < 0.01; +++*P* < 0.001.

Since MB21D2 is an annotated cadherin‐binding protein, we next assessed the impact of *MB21D2* overexpression and the Q311E recurrent mutation on cell migration and invasion which are cell–cell contact and cell‐matrix adhesion‐dependent processes. Wound‐healing assay using stable cell clones revealed accelerated migration in cells expressing WT and Q311E (Fig. 4A). Western blotting analysis (Fig. 4B, Fig. S8C) revealed enhanced expression levels of some markers triggered by WT or Q311E for epithelial–mesenchymal transition (EMT), including vimentin, CD44, phospho‐smad 2/3, Snail1, Twist 1/2 (in Q311E only), Bmi1 (WT only), and Slug (in Q311E only), in combination with downregulation of E‐cadherin. Consistently, our transcriptome data also revealed upregulation of those transcription factors involved in cancer stemness/EMT (Fig. S8D). To determine any cell morphology/phenotype changes associated with MB21D2 expression, we collected images of single cells at low cell density in collagen‐coated dishes. Both cellular and nuclear aspect ratios (major axis/minor axis) were significantly higher in CAL27 cells expressing WT and Q311E as compared with control cells with empty vector (Fig. 4C), suggesting the association of MB21D2 with highly migratory phenotypes. In addition, the Transwell invasion study showed that CAL27 cells expressing Q311E gained more migrative/invasive phenotypes, followed by cells with WT expression, while the control cells with empty vector showed much less invasiveness (Fig. 4D). To characterize cancer stemness potential activity, *in vitro* limiting dilution analysis was performed to measure the sphere‐forming efficiency (SFE) and cancer‐initiating cell (CIC) frequency of cells expressing the indicated constructs in suspension cultures. As shown in Fig. 4E and 4F, CAL27 cells expressing MB21D2 or its Q311E mutant showed higher stemness activity (WT: SFE: 47.5 % and CIC: 1/5.41; Q311E: SFE: 52.5 % and CIC: 1/9.35) than control cells expressing GFP (SFE: 5.0 %; CIC: 1/68.01). These data suggest that long‐term expression and selection of WT or Q311E MB21D2 trigger pro‐oncogenic activities by promoting EMT/cancer stemness.

### 2.5 Effects of MB21D2‐WT and Q311E mutant on the aggressiveness of xenografted tumors in nude mice model

**Fig. 5:**
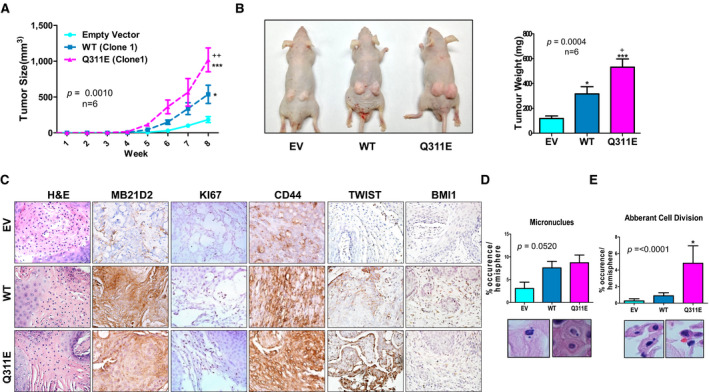
MB21D2 and its Q311E mutant mediate larger tumor murine xenograft model, upregulate stemness and proliferation marker, and increase genome instability. (A) CAL27 expressing different constructs were injected in nude mouse subcutaneously (3.5 × 10^6^). Tumor growth rates were monitored every seven days within 8 weeks after injection. Two‐way ANOVA and Tukey’s test of mean difference were carried out to determine significant difference in proliferation among groups. Data are presented as averages ± SD from six replicates. (B) Representative images of the mouse with tumor growth were taken (left‐hand panel), and tumor weights were determined after sacrifice (right‐hand panel). Data are presented as averages ± SD from six replicates (C) H&E (*n* = 5) and IHC staining (*n* = 3) was performed in the tumor xenograft section for the MB21D2, proliferation marker (KI67), cancer stemness cell marker (CD44), and cancer stemness associated with transcription factors (TWIST, BMI1) signal. (D) H&E staining (*n* = 5) was done to detect micronuclei formation and (E) aberrant cell division in mouse xenograft tumor section. Representative images (scaled bar 20 μm) were taken (upper panel) for micronuclei and aberrant cell division (lower panel) five independent experiments. For figure B, D, and E, one‐way ANOVA was used and mean difference among groups was validated using Tukey’s test. Statistical significance (EV control vs WT or Q311E): *P < 0.05; **P < 0.01; ***P < 0.001. (WT vs Q311E): ^+^ *P* < 0.05; ^++^ *P* < 0.01; ^+++^ *P* < 0.001.

To confirm that pro‐oncogenic phenotypes are mediated by *MB21D2* overexpression and Q311E mutation, we injected CAL27 (clones 1 and 2) and TW206 cells stably expressing MB21D2‐WT and its Q311E mutant into nude mice. A stable CAL27 cell clone with an empty vector served as control. Significantly, faster tumor growth and larger tumor sizes were observed in mice with Q311E expression in the xenografted tumors as compared to mice in WT and control groups (Figs. 5A,B). Although tumor lesions in the WT group did not grow as fast as the Q311E tumors, they still showed more aggressiveness as compared to tumors in the control group (Fig. 5A,B). Similar to the clone 1, the other CAL27 clone (clone 2) and TW206 stable cell lines with WT and Q311E expression are also more tumorigenic as compared to empty vector controls (Fig. S6B and S6C). IHC staining of tumor sections revealed stronger cancer stemness markers (anti‐CD44, Twist, and Bmi1) and higher proliferative activity (anti‐Ki67) in tumor lesions stably expressing WT or Q311E (Fig. 5C). Furthermore, those tumors showed higher events for micronucleus formation (Fig. 5D), suggesting genome instability triggered by WT or Q311E expression. In particular, aberrant cell division can be frequently found in tumors with stable Q311E expression, which is relatively rare in the other two groups (Fig. 5E). These data support the point of view that Q311E substitution in MB21D2 can trigger activation of pro‐oncogenic signaling and may bypass stress‐induced cell growth arrest/death, resulting in highly proliferative and genetically unstable cancer cells. On the other hand, long‐term expression of WT possibly selects clones with defects in stress sensing/responses that subsequently enrich cell populations with advantages in cell proliferation and genome instability.

### 2.6 MB21D2 and its Q311E mutant form act as enrichment factors for KRAS signaling and subsequently activate PIK3CA, AKT1, and CREB

**Fig. 6:**
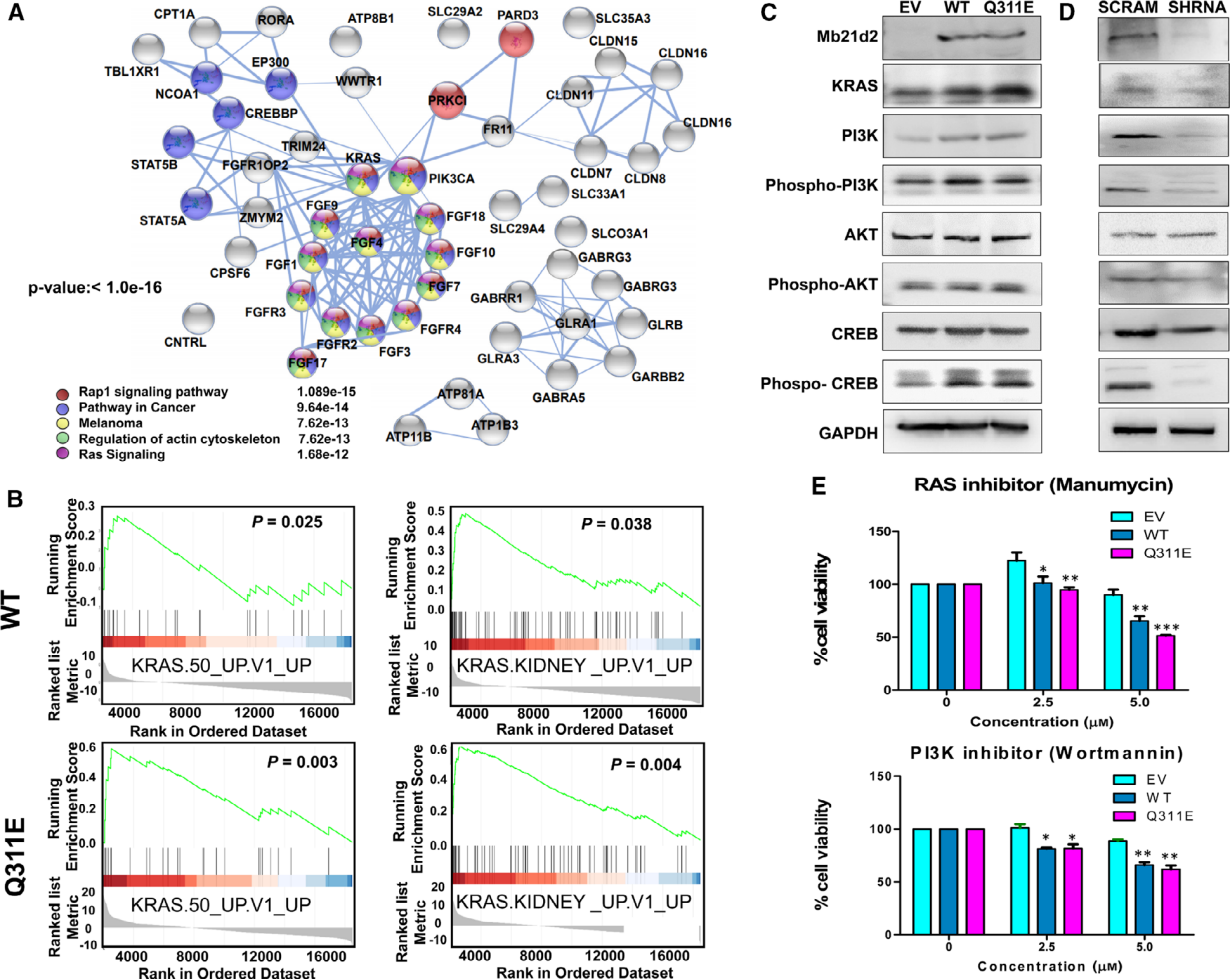
MB21D2 mediate enrichment and upregulation of KRAS activity and increase PI3K, AKT, and CREB phosphorylation. (A) mRNA sequencing data from TCGA were collected and analyzed using GSEA. Genes collected on the pathway enriched by WT MB21D2 (overexpression) based on gene set enrichment analysis (GSEA). (B) CAL27 stable cell line expressing different constructs (*n* = 2) was sent for RNA sequencing. Common pathway enriched by WT‐MB21D2 (upper panel) and its Q311E mutant (lower panel) using GSEA. (C) Western blotting of KRAS, PI3K, AKT, and CREB of CAL27 (*n* = 2) expressing different constructs. (D) Western blotting of KRAS, PI3K, AKT, and CREB of FADU (*n* = 2) transfected with anti‐MB21D2 shRNA and scrambled (control). (E) Drug responses (*n* = 6) of WT‐ and Q311E‐expressing stable cell lines vs EV control with known RAS inhibitor (manumycin) and PI3K inhibitor (wortmannin). Two‐way ANOVA validated by Tukey’s test of significance was carried out to determine significant difference in drug sensitivity among groups. Data are presented as averages ± SD of six replicates. Cell viability in response to 5 μm manumycin (right panel). Statistical significance (EV control vs WT or Q311E): *P < 0.05; **P < 0.01; ***P < 0.001. (WT vs Q311E): ^+^ *P* < 0.05; ^++^ *P* < 0.01; ^+++^ *P* < 0.001.

To determine possible pathways involved in tumor aggressiveness induced by WT and Q311E, we first utilized mRNA expression data from the TCGA cohort to perform gene set enrichment analyses (GSEA) and determined which pathways could be correlated with *MB21D2* overexpression. From pathways with statistical significance (*P*‐values < 0.05) (Table S3), we then selected genes in all pathways and used the STRING database (https://string‐db.org/) to determine protein–protein interactomes associated with *MB21D2* overexpression. As shown in Fig. 6A, the top‐five pathways are Rap1 signaling pathway (*q*‐value = 1.8e‐15), pathways in cancer (9.64e‐14), melanoma (7.62e‐13), regulation of actin cytoskeleton (7.62e‐13), and RAS signaling pathway (1.68e‐13). Interestingly, we found certain genes involved in the compact interactome that control those top‐five pathways, including KRAS, PIK3CA, FGF, and FGFR genes (Fig. 6A). To validate the key genes/pathways, we performed transcriptome sequencing and GSEA on our stable CAL27 cell clones. Our data showed positive enrichments of KRAS oncogenic signature commonly shared by both cell clones, either expressing WT or Q311E as compared to control cells (Fig. 6B, Table S4). Of note, these pathways became much more significant in cells expressing Q311E as compared to cells expressing WT, including KRAS.50_UP.V1_UP (*P* = 0.025 for WT and *P* = 0.003 for Q311E) and KRAS.KIDNEY_UP.V1_UP (*P* = 0.038 for WT and *P* = 0.004 for Q311E), indicating a functional relevance of this Q311E recurrent mutation.

Since both transcriptome data from clinical samples and data from engineered cells showed the association of MB21D2 overexpression with KRAS signaling, we then verified whether such enrichment can also be detected at the protein level. As expected, elevated KRAS was found in cells with WT and Q311E expression as compared to empty vector controls (Fig. 6C, Fig S8A). We then checked the downstream effectors known to be regulated by KRAS, such as PI3K, AKT, and CREB. Protein levels of PI3K and CREB were increased in WT and Q311E groups, which were associated with increased phosphorylation (Fig. 6C, Fig S8A). Though the protein levels of AKT were not changed by WT and Q311E expression, AKT was still activated by phosphorylation (Fig. 6C). Reversely, MB21D2 knockdown in FADU cells decreased total protein levels of KRAS and PI3K, along with dephosphorylation of PI3K, AKT, and CREB (Fig. 6D, Fig S8B). Cell line screening also showed that cells with high MB21D2 (FADU and HSC3 cells) expressed high levels of total and phosphorylated PI3K as compared to those with low MB21D2 expression (CAL27 and TW206 cells) (Fig. S4C). Since the KRAS‐PI3K axis was shown to be enriched/upregulated by WT or Q311E expression, we endeavored to know whether KRAS or PI3K reduction could be utilized as a strategy against MB21D2. We treated OSCC cells with RAS (manumycin) and PI3K (wortmannin) inhibitors and found higher chemosensitivity in cells expressing high MB21D2 (HSC3 and FADU cells) as compared to cells with lower MB21D2 (CAL27 and TW206) (Fig. S9A). In the engineered CAL27 and TW206 cells, we also confirmed that the overexpression of MB21D2 and its Q311E mutant made cells more sensitive to RAS and PI3K inhibitors as compared to empty vector controls (Fig. 6E and Fig. S9D and S9E). Through RAS inhibition, we can also see differential therapeutic effects on cells expressing WT or Q311E with limited effects on control cells (Fig. 6E). Our data suggest that WT MB21D2 overexpression and its Q311E mutation can positively influence the enrichment of KRAS to mediate aggressive cancer behavior. Anti‐KRAS could be utilized as a good strategy to develop new methods for treating cancers with MB21D2 overexpression or its Q311E recurrent mutation.

## 3 Discussion

Cadherin and its binders are critical for cellular and histological functions, particularly in squamous cells. In cancer, it has been accepted that cadherin functions either as a tumor suppressor or as a pro‐oncogenic protein by acting as a switch during epithelial–mesenchymal transition [40]. Given these biphasic roles of cadherin, its dependence on its binding partners could better clarify the pro‐oncogenic signature. Although the molecular functions of some cadherin binders such as beta‐catenin have been well‐addressed, we know very little about the roles of other potent binders in cancer development. In this study, we applied the 20/20 rule [12, 18] to discover other cancer‐associated cadherin binders. We discovered a unique recurrent Q311E mutation in *MB21D2* across human SCCs. *MB21D2* is overexpressed in various cancer types, including HNSCC, LUSC, ESCA, and CESC (Fig. S1A). Its overexpression in HNSCC correlates with poor clinical outcomes. Cell‐based assay revealed a significant increase in cell proliferation, migration, invasion, and *in vivo* tumorigenicity triggered by MB21D2 and its Q311E mutant by enrichment of KRAS signature that is known to subsequently activate the PI3K‐AKT pathway [41, 42]. To our knowledge, this is the first report to show the functional relevance of MB21D2 overexpression and its Q311E mutant as pro‐oncogenic proteins in HNSCC.

One consideration in HNSCC development is HPV infection. Based on our study, *MB21D2* overexpression and Q311E mutation correlated negatively with HPV infection, either by using E6/E7 viral marker or by using p16/CDKN2A host‐cell marker (Fig. S2). Since the NRG oncology HN‐002 study revealed that HPV + HNSCC responds favorably to cisplatin‐based radiation therapy [43], we tested any advantages in regulating chemosensitivity by treating cells with cisplatin and 5‐FU, two frequently used drugs for HNSCC. Our data indicated that cells expressing MB21D2 and its Q311E form can survive better than the control cells and still proliferate in the presence of cisplatin (the newly added Fig. S9B). However, such drug resistance effect was not detected in cells treated with 5‐FU (Fig. S9C). These data suggest that patients with *MB21D2* overexpression and Q311E mutation may show less sensitivity toward DNA‐damaging agents, such as cisplatin‐based radiation therapy. Nevertheless, anti‐RAS therapy could be a possible strategy to treat cancers with MB21D2 overexpression.

In this study, we noticed dramatically different data between transiently transfected cells and final stable clones with MB21D2‐WT expression. We therefore proposed MB21D2‐WT overexpression as a selection barrier/evolutionary driver to select and enrich cell clones with better survival advantages. Several well‐known oncogenes, for example, PI3K and RAS, can trigger cellular senescence or growth arrest frequently associated with DNA replication stress, DNA double‐strand breaks [44], and caspase‐independent cell death [45]. Oncogene‐induced senescence could promote cancer initiation and development through combined alteration of downstream effectors and the microenvironment, such as senescence‐associated inflammation [46]. The predicted interaction of MB21D2 with Mab21‐nucleotidyltransferase enzymes (Fig. S11B), such as cGAS, and mitochondrial regulator, such as MIEF proteins (Fig. S11B) [27, 28], may further explain the clonal selection event, as fittest clone selection was previously observed in many cancers such as multiple myeloma [47]. In our current study, we noticed that HNSCC samples with high MB21D2 levels correlated with low p16/CDKN2A expression (Fig. S2B and S2C), which is one of the critical genetic features for senescent cells to re‐enter the cell cycle and start cell proliferation by RAS expression/activation [48].

Prevalent hotspot mutations in *BRAF* (V600E), *KRAS* (G12D), and *PIK3CA* (E545K) are found in the functional domains [49] associated with oncogenic advantages. Consistent oncogenic activity of Q311E supported by transient and stable expression studies suggests gain of function of this recurrent mutation. However, a recent study also suggested that the Q311E recurrent mutation might be the result of its position at the DNA stem‐loop region, a favorable substrate for APOBEC3A [50, 51]. Notably, no evidence from those studies indicated the association between a DNA stem‐loop structure and protein function. We therefore tried to confirm the functional relevance of Q311E recurrent mutation in this study. Firstly, we found Q311E substitution in the Mab21 domain, a critical functional domain for Mab21 domain‐containing proteins (Fig. 1B). Secondly, this position is just nearby the phosphorylation site Y310 (Fig. S10A). In cGAS (a.k.a. MB21D1), the phosphorylation site (Y215) in Mab21 domain controls nuclear/cellular functions [52]. Structural prediction also revealed a local conformational change from a helix to a β‐sheet connecting to a long loop by Q to E substitution (Fig. S10B). Such conformational change may enable MB21D2 to stabilize the interaction with oncogenic effectors or complexes. Thirdly, transcriptome analyses confirmed the enrichment of KRAS signaling in MB21D2‐expressing cells and such oncogenic signaling became more enhanced in cells expressing Q311E mutation (Fig. 6B). Higher oncogenic activities by Q311E mutation can also be found as compared to MB21D2‐WT, including cell survival after transient transfection (Fig. S5), colony formation (Fig. 2B), and tumor growth/weight (Fig. 5A/5B and S6). Therefore, Q311E recurrent mutation should be functionally active that may mimic MB21D2 overexpression and possibly phosphorylation activation to enhance the downstream effectors involved in KRAS signaling.

Our results suggest that the WT and or Q311E mutant may serve as an enrichment factor for KRAS which is known to be a positive regulator of PI3K, AKT, and CREB signaling molecules (Fig. 6F). Our analysis of the genetic correlation between *MB21D2* and *PIK3CA* (including overexpression or mutation) in HNSCC patients revealed a strong association between these two genes (Fig. S12A and S12B), suggesting functional relevance between them. To confirm this, we searched for possible MB21D2‐interacting partners using mass–mass spectrometry data from BioGRID database (https://thebiogrid.org/). Surprisingly, the top‐six downstream interactome (*P* < 1.0E‐10) all contributed to Rab and Ras signaling (Fig. S11A). Although PI3K was not found as a direct binder for MB21D2, the interactome among Mab‐21‐containing proteins reveals that certain MB21D2‐interacting proteins may participate in activation of PI3K, such as ITPRIP (inositol 1,4,5‐trisphosphate receptor‐interacting protein; a.k.a. DANGER) and ITPRIPL1 (inositol 1,4,5‐trisphosphate receptor‐interacting protein‐like 1) (Fig. S11B) which are involved in IP3 signaling and calcium release/homeostasis[30, 53]. Thus, we consider that MB21D2 overexpression or its Q311E mutation can promote PI3K activation through KRAS signaling enrichment. Interestingly, MB21D2 upregulation correlated with PTEN downregulation signature (Table S4), which is more pronounced in Q311E as compared with WT. How MB21D2 and PTEN control the yin–yang balance in PI3K‐AKT signaling and contribute to cell transformation or cancer progression are under investigation.

The observed downregulation of E‐cadherin and upregulation of EMT markers (Fig. 4B) may further support the KRAS enrichment (Fig. 6A,B) which is a popular event in aggressive cancer types [54]. Transcriptome analyses revealed upregulation of several transcription factors involved in cancer stemness/EMT, including Twist 1/2, Slug, Snail1, and Bmi1, by MB21D2 overexpression or its Q311E mutant (Fig. S8D). These findings can be further validated by western blot *in vitro* and IHC staining *in vivo* (Fig. 4B and 5C). Some minor differences in transcriptional regulations on cancer stemness were found, such as Slug and Snail1 were enhanced only by Q311E, whereas Bmi1 was upregulated by WT‐MB21D2 (Fig. 4B and S8D). The mechanisms that determine the differential downstream effects between MB21D2 and its Q311E are still unknown and need further study. One of the possibilities would be that the conformational change in MB21D2 caused by Q to E substitution may alter the binding affinity toward certain downstream effectors.

In transgenic mice, *PIK3CA*‐activating mutation is insufficient to initiate tumorigenesis, and additional genetic alterations are required to drive this process, such as *TP53*/*PTEN* alteration [55]. It is highly possible that *MB21D2* overexpression or Q311E mutation serves as a secondary regulatory loop to control PI3K activity during cell transformation and cancer development through KRAS upregulation. Currently, we are preparing to perform genome editing in a mouse model to introduce the Q311E mutation, which will enable us to further validate the oncogenic potentials of the Q311E mutation in cancer. Nevertheless, our study suggests that overexpression of wild‐type MB21D2 and Q311E mutation mediates pro‐oncogenic activities in HNSCC by positive enrichment of KRAS along with activation of PI3K‐AKT, and EMT factors. Finally, we recommend the use of RAS inhibition as a new strategy for treating HNSCC with Q311E mutation and overexpression in MB21D2.

## 4 Materials and methods

### 4.1 Patient samples

China Medical University Hospital performed the patient tumor collection following written informed consent. The patients’ tumor was used to produce tissue array slide. This study involving patient tumors (tissue array) was approved by the China Medical University Hospital Institutional Board (CMUH102‐REC1009). This conforms with standards set by the Declaration of Helsinki.

### 4.2 Reagents and materials

Oral cancer cell lines used in the study, including CAL27 (from tongue), FADU (pharynx), HSC3 (tongue), and TW206 (tongue), were obtained from the Bio‐resource Collection and Research Center (BCRC), Taiwan. Those cell lines used in the study are HPV negative [56, 57]. FADU cell line carries the R248L hotspot mutation, and no hotspot mutation was found in other cell lines [58]. All cells were supplemented with DMEM containing 10% FBS and 1% penicillin/streptomycin mixture (Gibco/Thermo Fisher Scientific, Waltham, MA). Cells were tested for mycoplasma by mycoplasma‐specific PCR kit (cat#BSMP101) and DAPI (cat#D1306) staining prior to usage. Human MB21D2 cDNA clone was purchased from transOMICS Technologies (Clone No. BC045582; Genome Way Northwest, Huntsville, AL, USA). Restriction enzymes EcoR1, BamH1, and DPN1 were purchased from New England BioLabs (Bâtiment 6 5 rue Henri Desbruères 91030 EVRY Cedex, France). Anti‐MB21D shRNA(CCTGGACTTAGATGAGCTTAACCGGCCTGGACTTAGATGAGCTTAACTCGAGTTAAGCTCATCTAAGTCCAGGTTTTTTG) was purchased from RNAi core facility of Academia Sinica, Taiwan (http://rnai.genmed.sinica.edu.tw/), and selected based on validation testing. The details of the antibody and primers used in this study are listed in Table S6.

### 4.3 Recurrent mutation and gene expression screening by data mining

To screen for recurrent mutation in known and putative cadherin‐binding genes, we searched the genes from the UniProt database by using function‐based annotation. All the genes were profiled for total mutation and recurrent mutation rates using The Cancer Genomic Atlas (TCGA) databank (https://www.cbioportal.org/) [59, 60]. All genes with mutation rates ≥ 2% were selected for recurrent mutation analysis. Expression assessment and patient survival analysis were performed on the gene with the highest recurrent mutation rate (mutation events at a specific site of total events) using the cBioportal and GEPIA (http://gepia.cancer‐pku.cn/) databases [61].

### 4.4 Immunohistochemistry (IHC), H&E staining, and western blotting

Normal and tumor (tissues array), and mouse xenograft were processed for IHC and H&E staining, according to the protocol as described previously [62].

### 4.5 Subcloning and mutagenesis

The cDNA clone of MB21D2 was amplified (see primer sequence at Table S6) and subcloned in pEGF‐N1 (Clontech, Mountain View, CA, USA). EcoRI (NEB#R3101) and BamHI (NEB#R3136) served as the in‐frame cloning site. Mutagenesis was done using conventional overlap extension PCR [63]. Sowed fragments were digested using the designated restriction enzyme. Ligation was done using the Thermo Fisher Ligation Kit (cat#K1422). Digestion using DPN1 (NEB# R0176S) was done before ligation to ensure that there was no old template degradation before transformation. Positive clones were submitted for DNA sequencing to obtain desired clones (Fig. S13).

### 4.6 Overexpression, knockdown, and stable line generation

Cells (CAL27 and TW206) were transfected using Lipofectamine 2000 with 3000 ng·µL^−1^ of the following: empty vector (control), wild‐type MB21D2 (WT), and Q311E constructs. Cells were allowed to recover after 6 h using penicillin/streptomycin‐free DMEM (with FBS), and the medium was replaced with DMEM (PS + FBS) after 12 h. Transfected cells were used for the experiment accordingly. *Stable line generation* was done in transfected CAL27 and TW206 cells by re‐seeding in 96‐well plate using DMEM containing G418 (700 μg·mL^−1^) at a cell density of 1 cell per well to obtain a single clone. After two weeks, clones were screened using fluorescent microscopy. Positive clones were subjected to expansion. Stable clones were confirmed using qPCR and western blot.

### 4.7 Cell‐based functional assay

*Cell proliferation* was carried out by MTT assay for four days, as previously described [62]. *Colony formation assay*. Cells were seeded in a 6‐well plate with a density of 500 cells/well and incubated for 7 days. Plates were fixed using 4% formaldehyde and subsequently stained with Crystal Violet for 1 h. Plates were rinsed to remove the excess stain to make colonies more visible. Plates were viewed using CLUBIO GC1160 viewer; colonies were quantified, accordingly. *Sphere Formation Assay*. Cells were re‐seeded in 24‐well plate containing 10% matrix gel DMEM (with 10% FBS, 1% PS), then overlaid with 40% matrix cell, and fed with fresh medium for 15 days. Sphere number and size were determined by microscopy at 10X magnification under DIC. *Wound healing, in vitro cell migration, and invasion and anti‐anoikis assay* were done as described previously [62, 64]. Modification in detecting of invading cells was done. Green fluorescence detection was done for transiently transfected cells, while DAPI staining was used for stable cell line invasion detection at 10X magnification. Potential cancer stemness activity was carried out by culturing cells in a Costar 96‐well ultra‐low/no adherent culture (Kennebunk, ME, USA) plates in a cell density of 1000, 100, 10, and 1 cell per well. Sphere formation was observed after 14 days. Intact shiny spheres were counted and measured accordingly.

### 4.8 Cell death/apoptosis assay

Transfected cells were gently washed and trypsinized. Cells were collected, and trypsin was immediately neutralized with DMEM. Cells were centrifuged and pelleted. Annexin V staining was done using Annexin V‐FITC Apoptosis Staining/ Detection Kit (cat#ab14085). Fluorescence microscopy was carried out to analyze cell death/apoptosis.

### 4.9 Cell and nucleus aspect ratio analysis

Approximately 100 cells (stable CAL27) were seeded in a glass‐bottom dish coated with collagen. After 24 h, cells were stained with DAPI. Single cells were measured for the cell aspect ratio using the DIC filter and nucleus aspect (major axis/minor axis) ratio using blue filter. 30 single cells from each group were considered for cell and nucleus ratio analysis.

### 4.10 Transcriptome sequencing

RNA was extracted using the QIAGEN RNeasy Mini Kit (Cat No./ID:74104). Quality control was done by Gel Checking and Absorbance 260/280 using NanoDrop. Samples were submitted for transcriptome sequencing using HiSeq 2000 (Illumina NovaSeq 6000). Analysis was done by the service provider (Novogene). GSEA was used to determine enrichment pathways in wild‐type and Q311E mutant MB21D2 to determine common enriched pathways.

### 4.11 In vivo tumor formation (nude mouse xenograft study)

A total of 36 male NU/NU mice, aged 8–10 weeks purchased from BioLASCO Taiwan Co., Ltd., were used in the study. Using 18G x 11/2 Terumo needle, CAL27 (clone 1 mice: *n* = 6/group, clone 2 mice: *n* = 4/group), and TW206 (mice: *n* = 5/group) stably expressing control (EV), WT and Q311E mutant were subcutaneously injected into the mice with approximately 3.5 × 10^6^ cells in each injection site. Measurement was done every 7 days, and tumors were collected and weighed on the 8th week.

### 4.12 Drug sensitivity test

Drug sensitivity test was carried out by seeding 3000 cells per well. After 24 h, specific drug concentration of RAS inhibitor (manumycin), PIK inhibitor (wortmannin, CAS 19545‐26‐7, Santa Cruz Biotechnology, Dallas, TX, USA), and cisplatin and 5‐FU (Merck Darmstadt, Germany) were added accordingly. MTT assay was used to measure cell viability. All animal studies were blinded and conducted following the guidelines according to the Laboratory Animal Use of Kaohsiung Veterans General Hospital with a protocol approved by the Animal Study Committee (VGKHS‐2019‐A019).

### 4.13 Western blotting

Cells attaining around eighty percent (80%) confluence in 6‐well plates were collected using RIPA lysis buffer containing a cocktail of proteases and phosphatase inhibitor. The lysate was centrifuged at 14 000 ***g*** for 15 min to obtain the necessary proteins. BCA kit was used to determine and normalize the concentration of proteins. Proteins were run in 10% SDS/PAGE electrophoresis and blotted in PDVF membrane. Proteins of interest were probed using specific primary antibody and appropriate secondary antibody.

### 4.14 Statistical analyses

Statistical analysis was carried out using spss V.14.0 software (SPSS Inc., Chicago, IL, USA) and graphpad prism (GraphPad Software, La Jolla, CA, USA). t‐Tests and one‐way analysis of variance (ANOVA) were done to determine the significance of differences among groups, while two‐way ANOVA was used to determine the significance of differences with the group and time interval factors. Tukey’s test for significant difference was used for multiple comparison. Chi‐square was utilized for gene association study.

## 5 Conclusion

In our study, we found that MB21D2 recurrent mutation is highly frequent in SCCs, particularly in HNSCC. Overexpression of MB21D2 is correlated with shorter patient survival and promotes oncogenic advantages, such as cell proliferation, survival, and tumorigenicity based on vitro and nude mouse studies. Further, MB21D2 (WT overexpression or its Q311E mutation) participates in migration/invasion as reflected in EMT upregulation and phosphorylation. Consequently, we conclude that *MB21D2* overexpression and its Q311E recurrent mutation play an important role in HNSCC by acting as an enrichment factor of KRAS‐mediated signaling along with PI3K‐AKT and CREB and EMT activation. The negative association/correlation of MB21D2 with HPV may further explain the resistance of MB21D2 (WT or Q311E)‐overexpressing cells to DNA‐damaging drugs, such as cisplatin. On the other hand, the use of RAS inhibitor in MB21D2‐overexpressing cells could be a new or additional strategy in treating HNSCC that is resistant to DNA‐damaging agents.

## Conflict of interest

The authors declare no conflict of interest.

## Author contributions

Gracilla DE, Sheu JJC, and Korla PK conceptualized and designed the experiment. Gracilla DE performed the experiment. Gracilla DE, Sheu JJC, and Lai MT performed data analysis and wrote the manuscript. Sheu JJC, Lai MT, Chiang AJ, and Liou WS reviewed and edited the manuscript. All authors have read and agreed to publish the paper on this version.

## Supporting information


**Fig S1.** Expression of MB21D2 in all cancer types, HNSCC subtypes and expression of Q311E mutation in HNSCC.
**Fig S2.** Comparison and correlation of HPV and MB21D2 in HNSCC patients.
**Fig S3.** Stable clone verification.
**Fig S4.** Cell line profiling.
**Fig S5.** Transient transfection data.
**Fig S6.** Activity of WT‐MB21D2 and Q311E form in CAL27(clone 2) and TW206 clone.
**Fig S7.** Effects of MB21D2 knockdown on cell proliferation.
**Fig S8.** Relative intensity of probe proteins and mRNA expression of EMT markers.
**Fig S9.** Drug responses of WT MB21D2 and Q311E expressing cell lines.
**Fig S10.** Mutation profile and structural prediction of MB21D2 and its Q311E form.
**Fig S11.** Known and predicted interaction of MB21D2.
**Fig S12.** Association between MB21D2 overexpression and Q311E mutation with PIK3CA overexpression and mutation from actual patient sequencing data.
**Fig S13.** Cloning of wild‐type MB21D2 and Q311E form.

**Table S1.** Mutation rates of genes (312), annotated as cadherin binding (from UniProt database) based on 1490 patients, in four squamous cell carcinoma, namely, cervical squamous carcinoma (CESC), and esophageal squamous cell carcinoma (ESCA), lung squamous cell carcinoma (LUSC), and head and neck squamous cell carcinoma (HNSCC) obtained from TCGA sequencing databank.
**Table S2.** Cadherin Binding Genes with ≥ 2% mutation rate in 1490 patients in four squamous cell carcinoma namely cervical squamous carcinoma (CESC), esophageal squamous cell carcinoma (ESCA), lung squamous cell carcinoma (LUSC), and head and neck squamous cell carcinoma (HNSCC) collected from TCGA sequencing databank.
**Table S3.** Reactome and Genes Enriched by MB21D2 overexpression in clinical sample from TCGA sequencing data.
**Table S4.** Positively correlated pathway/signature enriched by MB21D2(Wild‐type and Q311E) in stable cell clones based on transcriptome sequencing.
**Table S5.** Co‐occurrence of MB21D2 with known PIK pathways regulators in HNSCC based on TCGA sequencing data.
**Table S6.** List of Primers and antibody dilution used in the study.

## Data Availability

All clinical data used in the study were obtained from TCGA data (cbioportal.org/) (head and neck squamous carcinoma). Accession number of MB21D2 gene and protein sequence is NM_178496.4. Transcriptome raw data sequence reads are available at NCBI (SRA Accession: PRJNA656896 ID:656896). Other data can be requested from the corresponding author upon reasonable request.
