# Supplementary material for: Overexpression of wild type or a Q311E mutant MB21D2 promotes a pro‐oncogenic phenotype in HNSCC
Source: Mol Oncol. 2020 Oct 15;14(12):3065–82. doi: 10.1002/1878-0261.12806 (PMC7718949; doi:10.1002/1878-0261.12806)
Supplement: Supplementary file 1 — Fig S1. Expression of MB21D2 in all cancer types, HNSCC subtypes and expression of Q311E mutation in HNSCC. Fig S2. Comparison and correlation of HPV and MB21D2 in HNSCC patients. Fig S3. Stable clone verification. Fig S4. Cell line profiling. Fig S5. Transient transfection data. Fig S6. Activity of WT‐MB21D2 and Q311E form in CAL27(clone 2) and TW206 clone. Fig S7. Effects of MB21D2 knockdown on cell proliferation. Fig S8. Relative intensity of probe proteins and mRNA expression of EMT markers. Fig S9. Drug responses of WT MB21D2 and Q311E expressing cell lines. Fig S10. Mutation profile and structural prediction of MB21D2 and its Q311E form. Fig S11. Known and predicted interaction of MB21D2. Fig S12. Association between MB21D2 overexpression and Q311E mutation with PIK3CA overexpression and mutation from actual patient sequencing data. Fig S13. Cloning of wild‐type MB21D2 and Q311E form. [file MOL2-14-3065-s001.pdf]

Supplementary Figures.

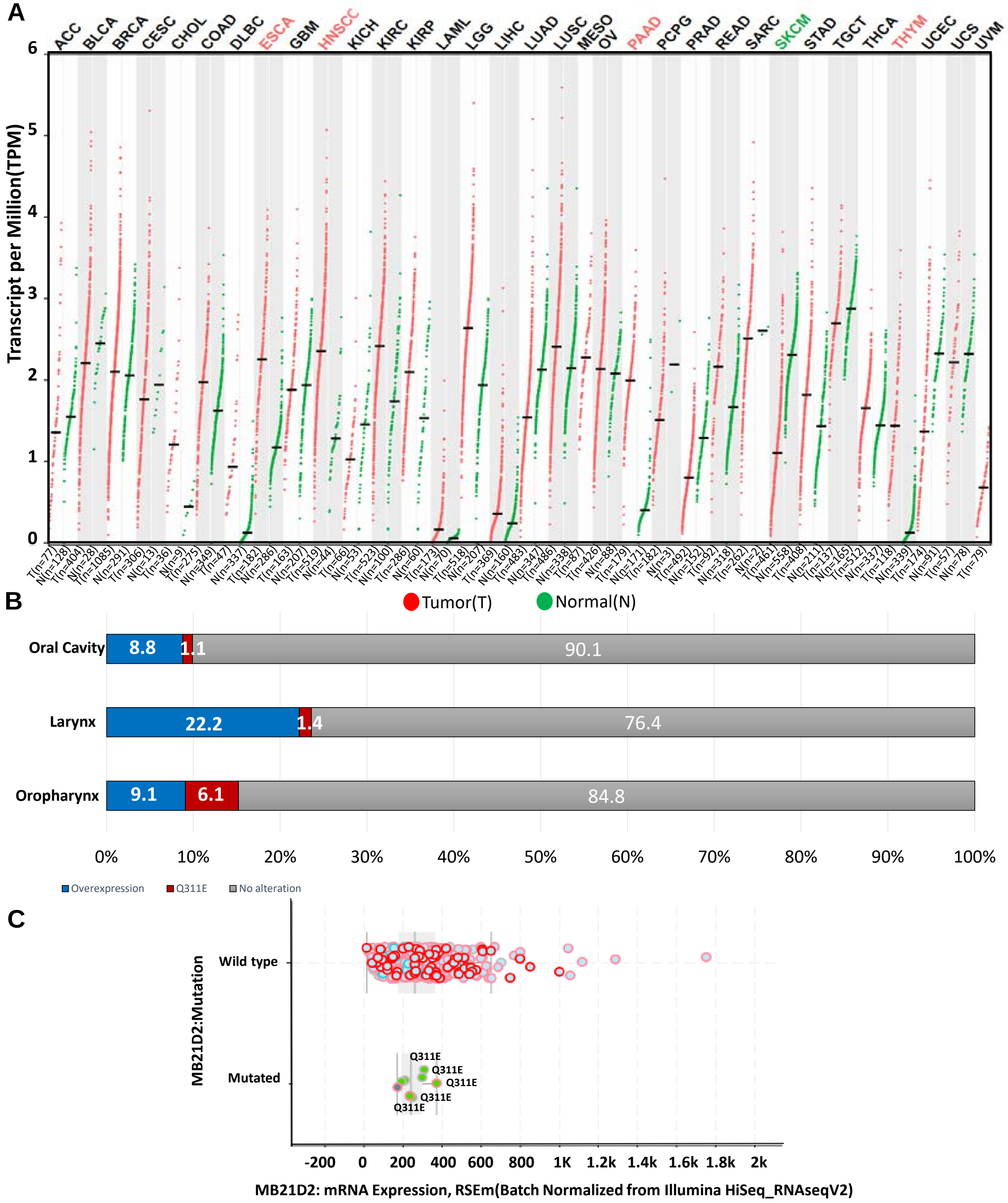

Figure S1. Expression of MB21D2 in all cancer types, HNSCC subtypes and expression of Q311E mutation in HNSCC. **A**. MB21D2 expression profile across several cancer types: ACC (adenoid cystic carcinoma), BLCA (bladder urothelial carcinoma), BRCA (Breast invasive carcinoma), CESC (Cervical squamous cell carcinoma and end cervical adenocarcinoma), CHOL (Cholangiocarcinoma), COAD (Colon adenocarcinoma), DLBC (Diffuse large B-cell lymphoma), ESCA (Esophageal carcinoma), GMB (Glioblastoma multiforme), HNSCC (Head and Neck squamous cell carcinoma), KICH (Kidney

Chromophobe), KIRC (Kidney renal clear cell carcinoma), KIRP (Kidney renal papillary cell carcinoma), LAML (Acute Myeloid Leukemia), LGG (Low Grade Glioma), LIHC (Liver hepatocellular carcinoma), LUAD (Lung adenocarcinoma), LUSC (Lung squamous cell carcinoma), MESO (Mesothelioma), OV (Ovarian serous cystadenocarcinoma), PAAD (Pancreatic adenocarcinoma), PCPG (Pheochromocytoma and Paraganglioma), PRAD (Prostate adenocarcinoma), READ (Rectum adenocarcinoma), SARC (Sarcoma), SKCM (Skin Cutaneous Melanoma), STAD (Stomach adenocarcinoma), TGCT (Testicular Germ Cell Tumors), THYM (Thymoma), THCA (Thyroid carcinoma), UCS (Uterine Carcinosarcoma), UCEC (Uterine Corpus Endometrial Carcinoma), UVM (Uveal Melanoma). Significant upregulation was found in Head and Neck Cancer, Pancreatic Adenocarcinoma, Esophageal Squamous Carcinoma and Thymoma. **B.** MB21D2 expression across several Head and Neck Cancer Sub-types. **C.** mRNA expression RSEM (Batch Normalized from Illumina HiSeq_RNAseqV2) of WT MB21D2 and its **Q311E** form in actual patient samples from TCGA data bank (https://www.cbioportal.org/) (right panel).

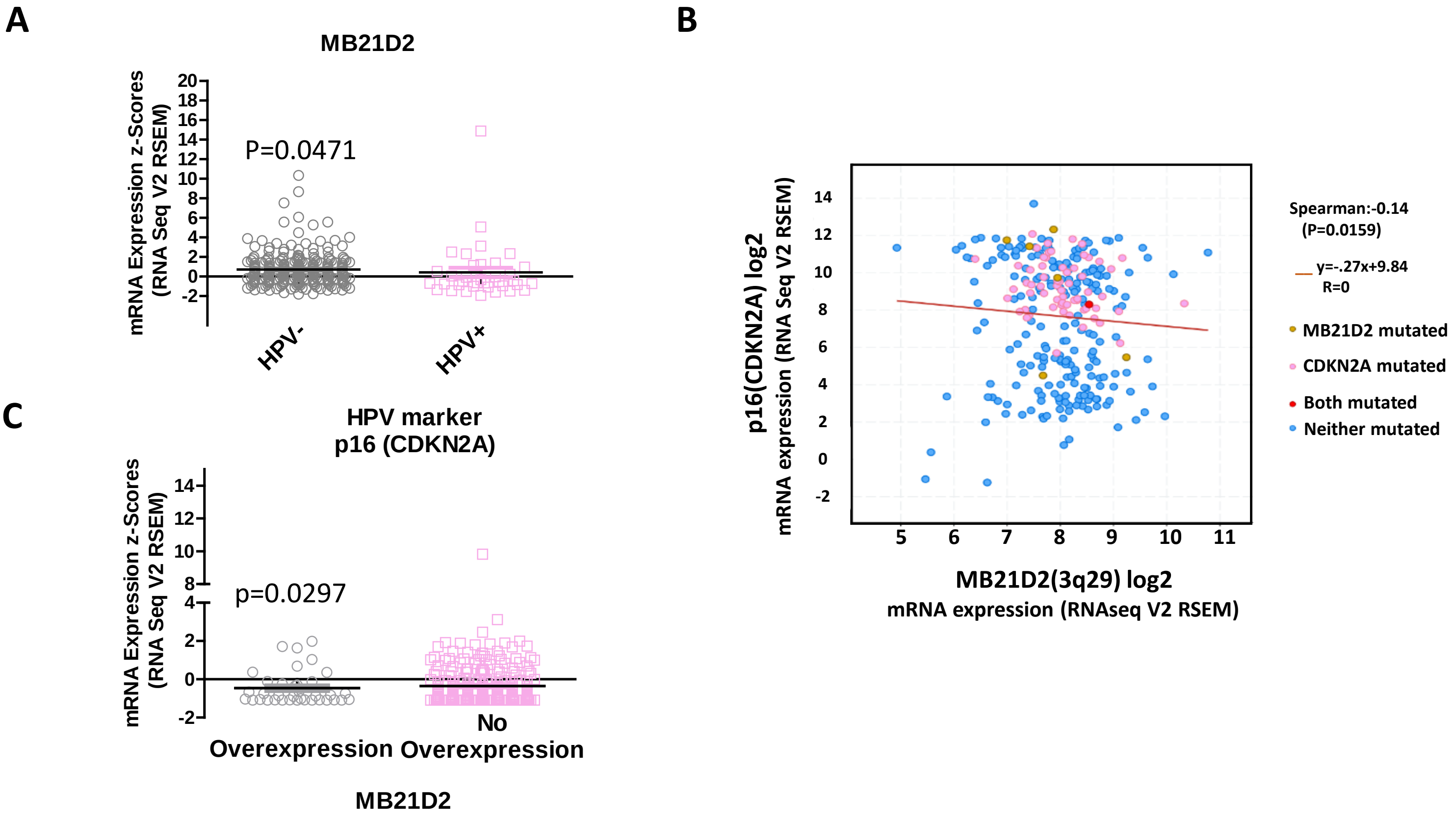

Figure S2. Comparison and correlation of HPV and MB21D2 in HNSCC patients. **A.** Comparison of MB21D2 expression in HPV+ and HPV- patients. **B**. Correlation between MB21D2 between p16(CDKN2A). **C**. p16 (HPV marker) expression in high-MB21D2 and Non-overexpressing MB21D2 patients.

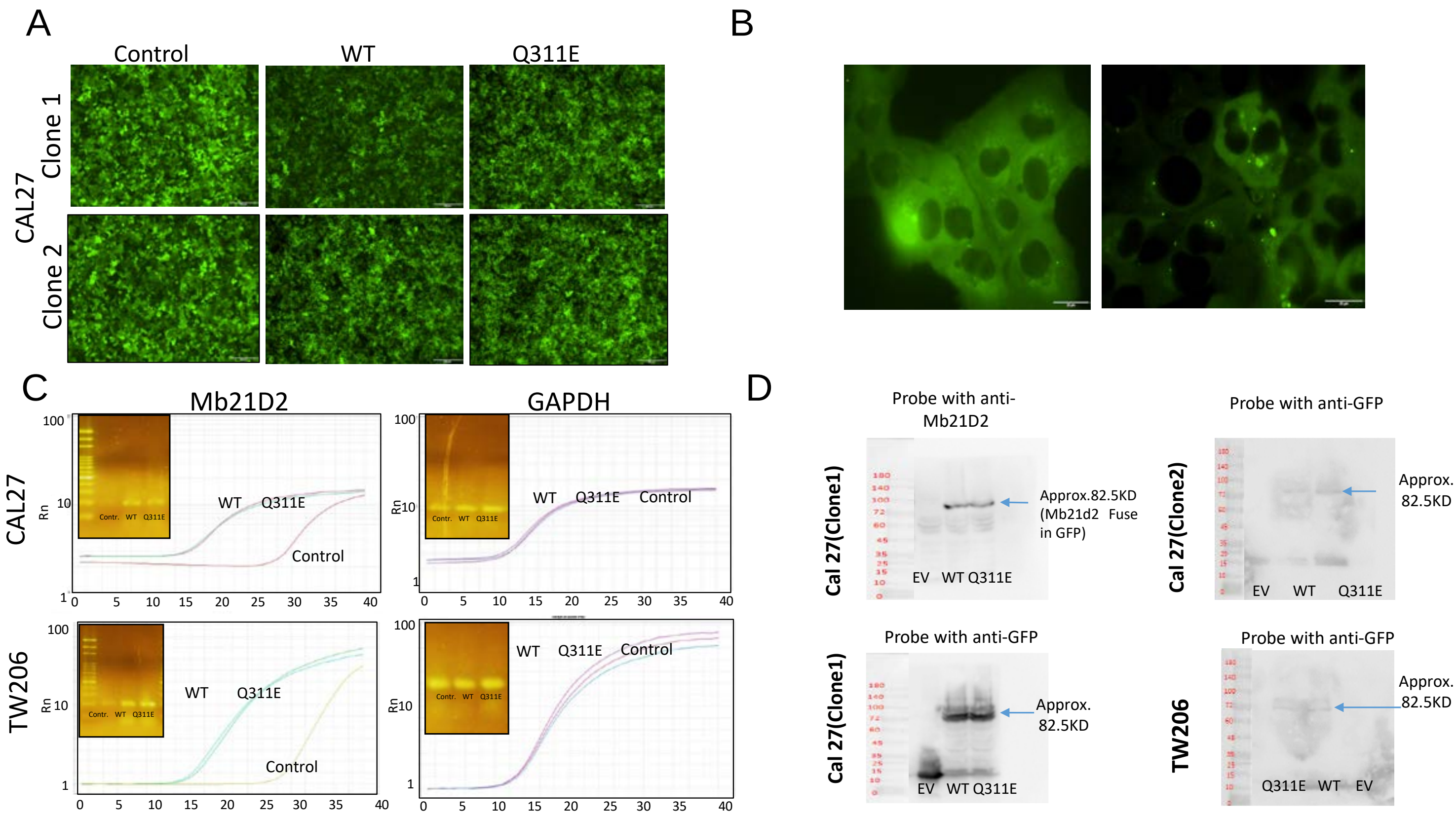

Figure S3. Stable clone verification. **A.** Fluorescence Micrograph of Cal27 (clone 1 and 2) cells stably expressing GFP, WT-MB21D2, and Q311E-MB21D2. **B.** Localization of MB21D2 in the Cal27 cells. **C.** mRNA expression of MB21D2 and its Q311E form in Cal27 and TW206 cells. **D.** Stable expression of MB21D2 and its Q311E mutant on the protein level.

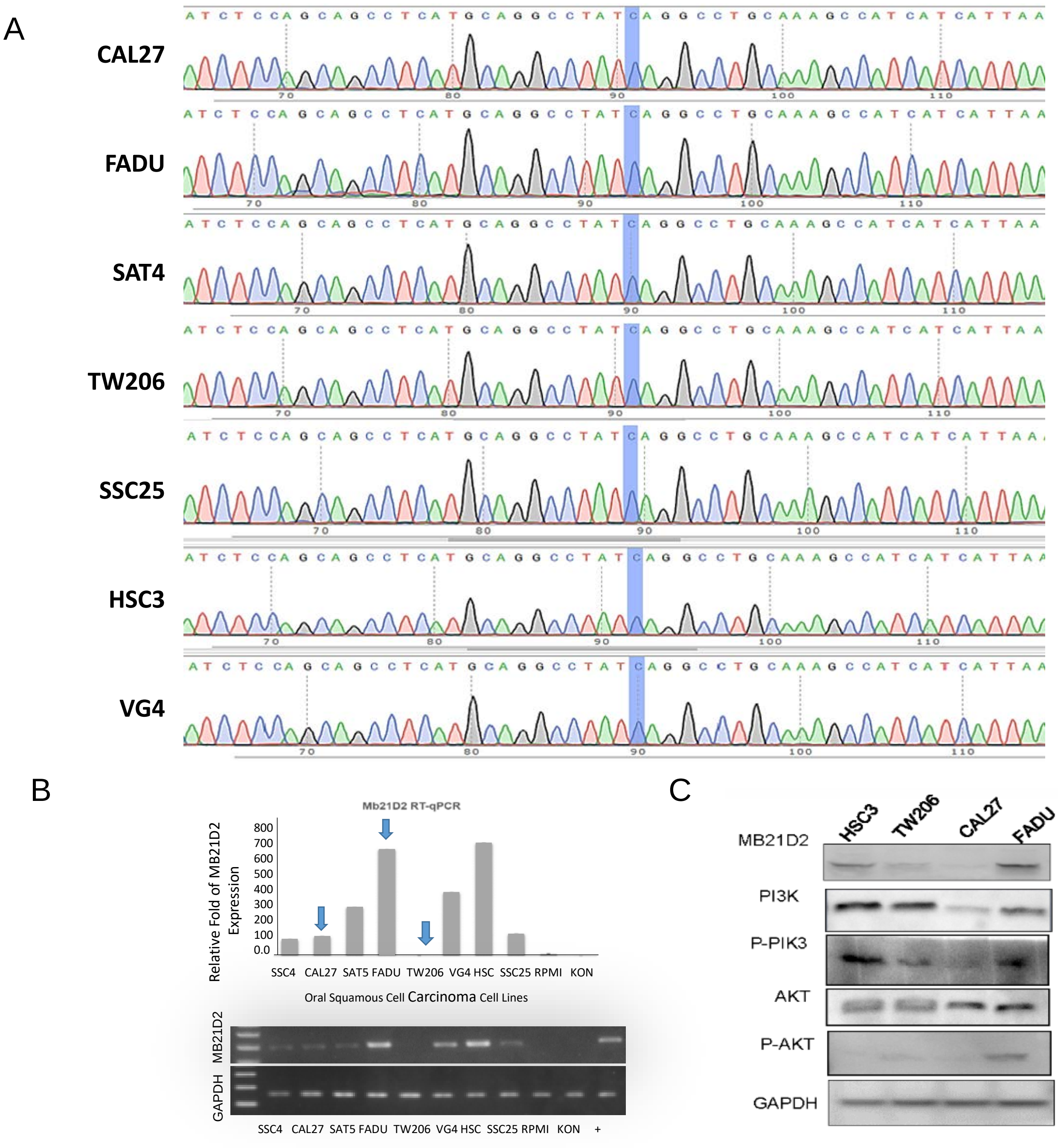

Figure S4. Cell line profiling. **A.** Genomic Sequences of MB21D2 showing the wild-type form in different OSCC cell lines. **B.** Expression profile of OSCC cell lines based on relative MB21D2 - mRNA expression. **C.** Protein expression profile of different OSCC parental cell lines.

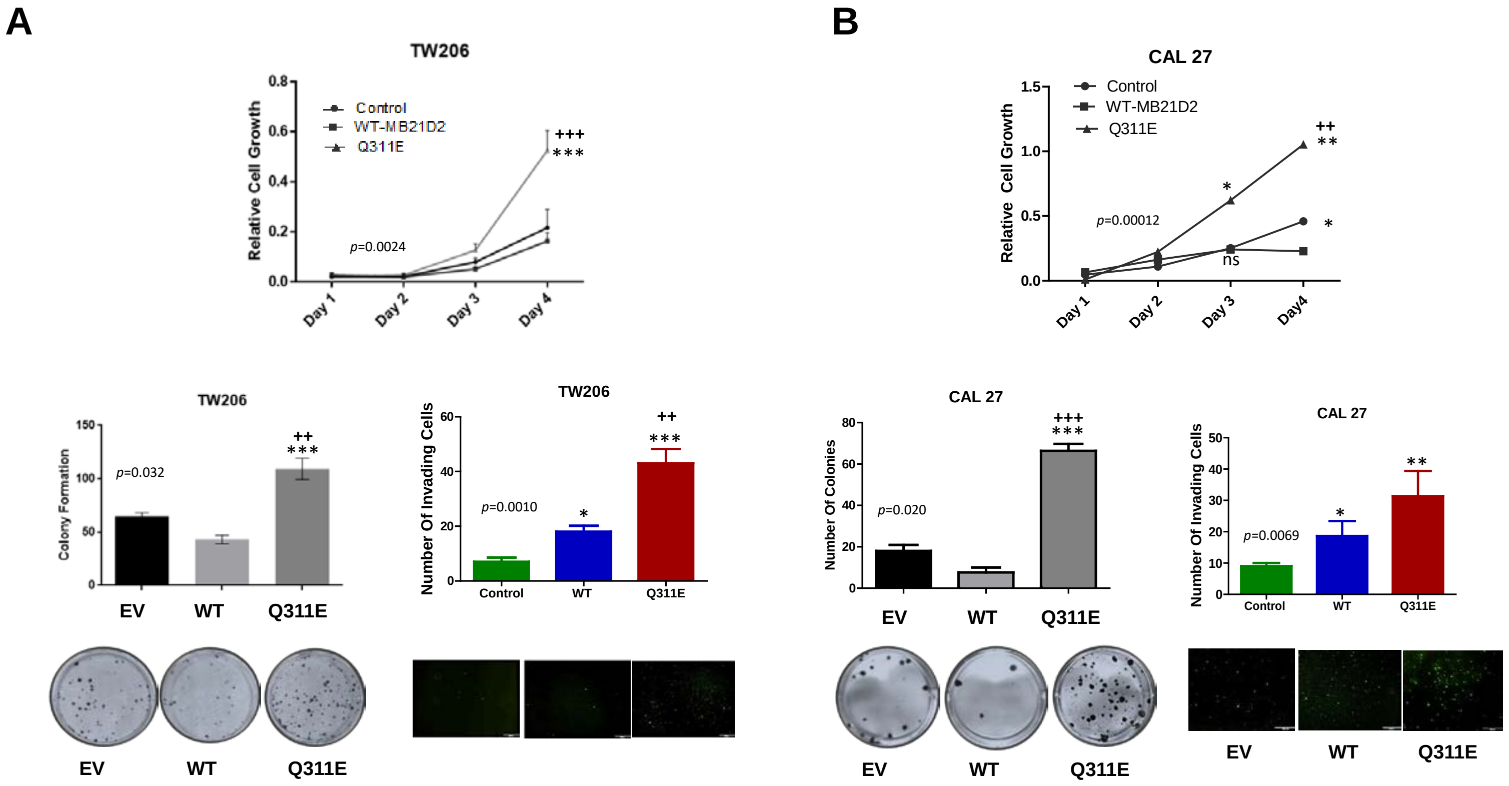

Figure S5. Transient transfection data. **A.** TW206 Proliferation (left upper panel), colony formation (left lower panel), invasion/migration (right upper panel). **B**. Cal 27 Proliferation (upper panel), colony formation (left lower panel), and invasion/migration (right lower panel). Statistical significance (EV control vs WT or Q311E): *, $p < 0.05$; **, $p < 0.01$; ***, $p < 0.001$. (WT vs Q311E): $^{+}$, $p < 0.05$; $^{++}$, $p < 0.01$; $^{+++}$, $p < 0.001$; ns: not significant.

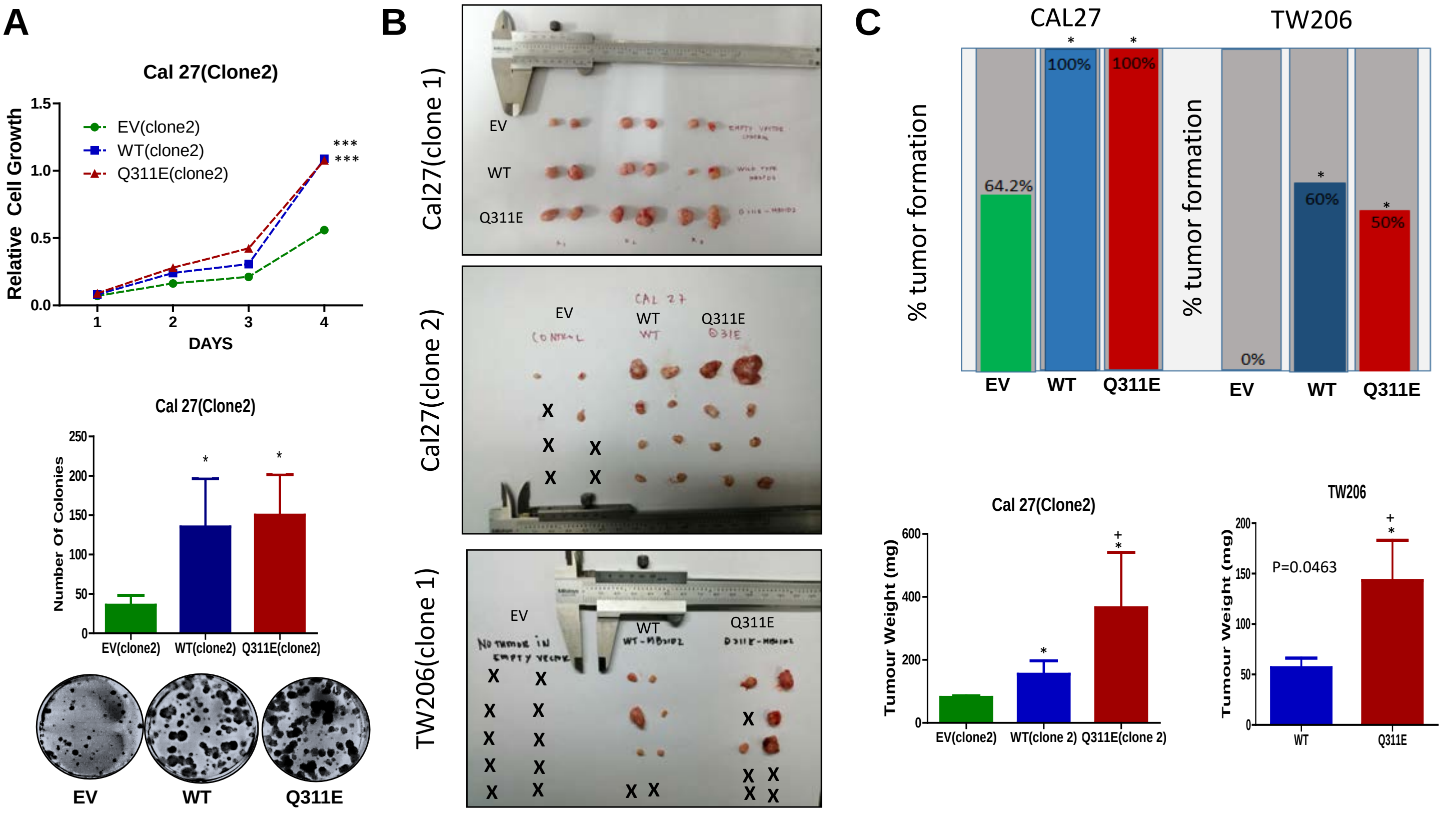

Figure S6. Activity of WT-MB21D2 and Q311E form in CAL27(clone 2) and TW206 clone. **A.** Proliferation (upper panel) and colony formation (lower) of CAL27 stable clone 2. **B.** Collected tumors from mouse xenograft of CAL27 cells (clone 1 and 2) and TW206 (lower panel)**.** **C** Percent of tumor formation in combined Clone 1 and 2 in Cal 27 stable cell lines and Tw206 (upper panel), tumor weight (lower panel). Statistical significance: *, $p < 0.05$; **, $p < 0.01$; ***, $p < 0.001$; ns = not significant. (WT vs Q311E): $^{+}$, $p < 0.05$; $^{++}$, $p < 0.01$; $^{+++}$, $p < 0.001$; ns: not significant.

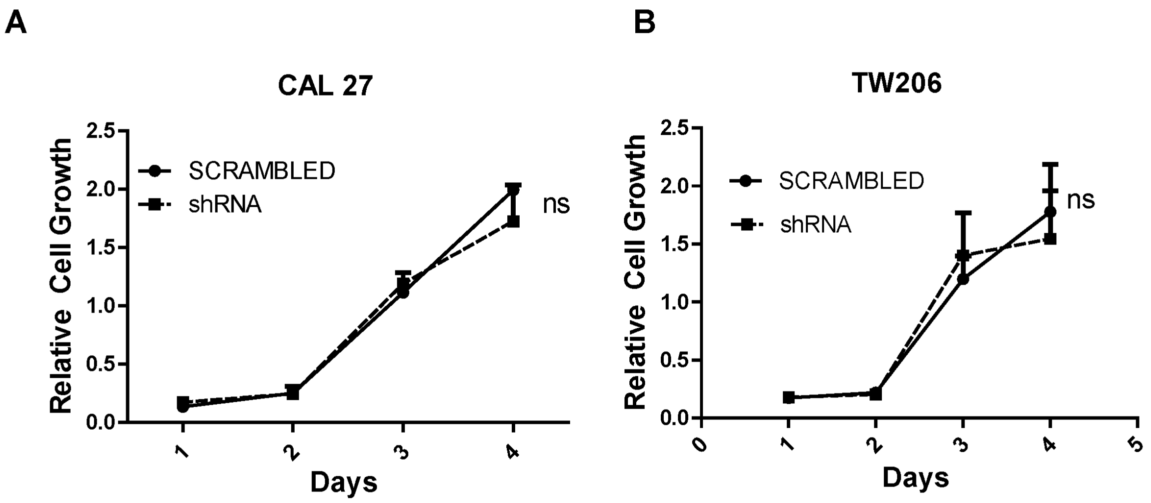

Figure S7. Effects of MB21D2 Knockdown on cell proliferation. **A**. Cal27 (low endogenous MB21D2) and **B.** TW206 (low endogenous MB21D2). Statistical significance: *, $p < 0.05$; **, $p < 0.01$; ***, $p < 0.001$; ns = not significant. (WT vs Q311E): $^{+}$, $p < 0.05$; $^{++}$, $p < 0.01$; $^{+++}$, $p < 0.001$; ns: not significant

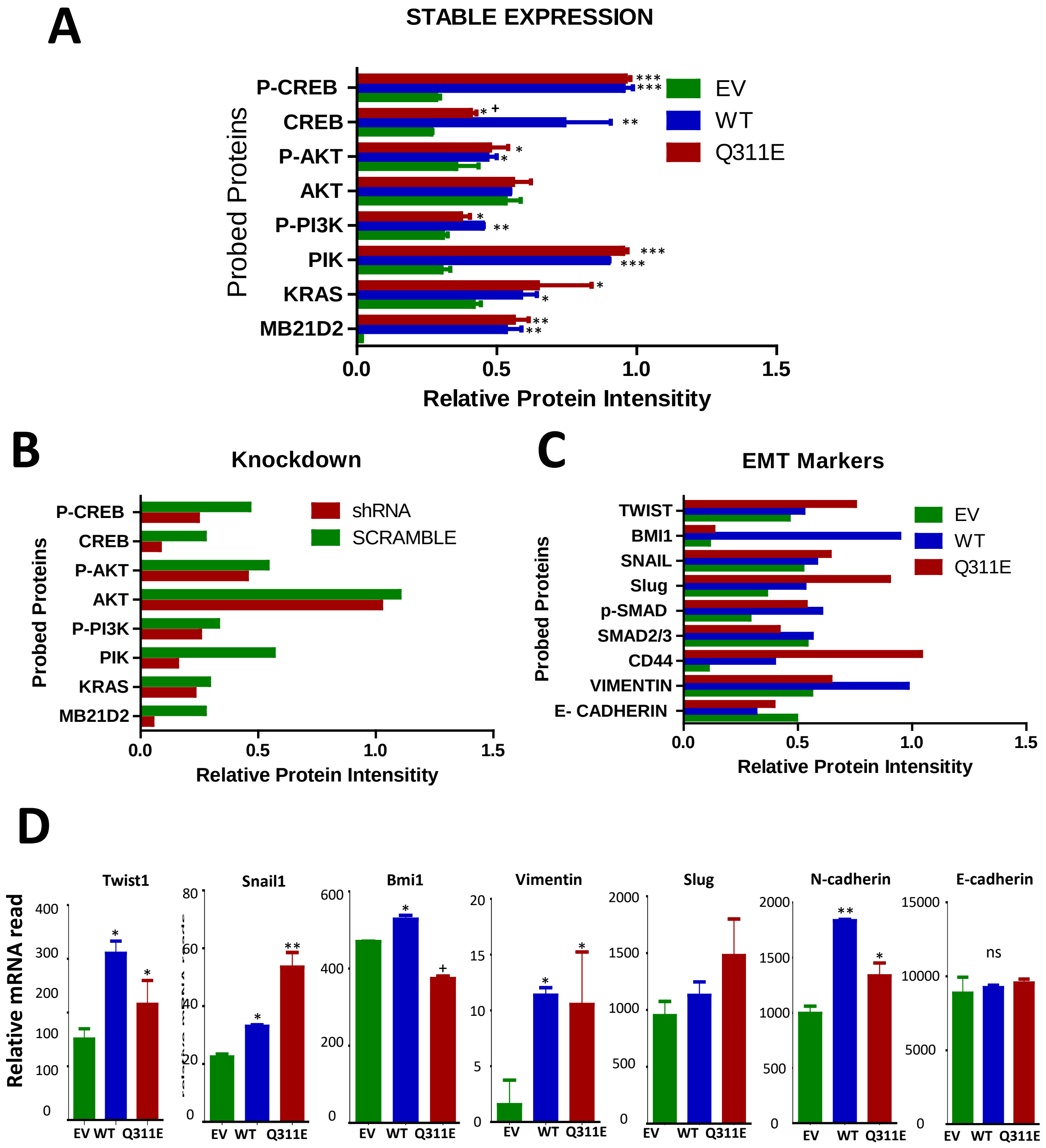

Figure S8. Relative intensity of probe proteins and mRNA expression of EMT markers. **A.** Stable line overexpression experiment (constitutive expression) in Cal 27. **B.** Knockdown experiment in FADU cell line. **C.** EMT markers in stable cell line overexpression experiment (constitutive expression) in Cal 27. **D.** Relative mRNA expression/reads of EMT markers based on transcriptome sequencing. Statistical significance: *, $p < 0.05$; **, $p < 0.01$; ***, $p < 0.001$; ns = not significant. (WT vs Q311E): $^{+}$, $p < 0.05$; $^{++}$, $p < 0.01$; $^{+++}$, $p < 0.001$; ns: not significant.

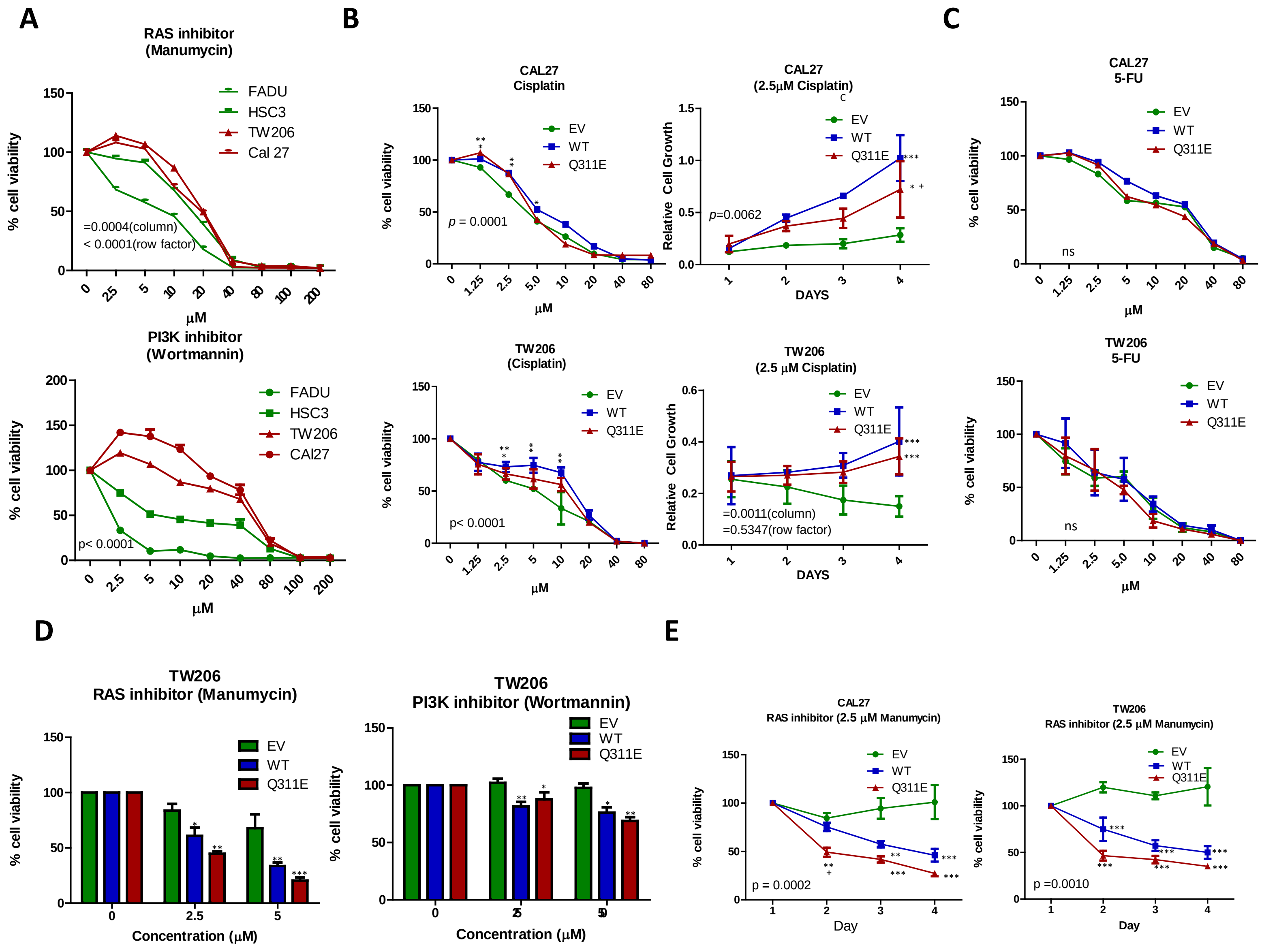

Figure S9. Drug responses of WT MB21D2 and Q311E expressing cell lines. **A.** Drug response of high and low MB21D2 expressing cells to RAS (Manumycin sc-20085 Santa Cruz Biotechnology) and PI3K inhibitor (Wortmannin, CAS 19545-26-7, Santa Cruz Biotechnology). **B.** Drug response of CAL27 and TW206 (expressing WT and Q311E MB21D2) to Cisplatin (Merck, Darmstadt, Germany) at different concentrations (left panel), response of TW206 at 2.5µM. **C.** Cell Growth Response of CAL27 and TW206 (expressing WT and Q311E MB21D2) to 5-FU (Merck, Darmstadt, Germany). **D.** Response of TW206 (expressing WT and Q311E MB21D2) to RAS inhibitor and PI3K inhibitor at 2.5 and 5.0µM. **E.** Cell growth of CAL27 TW206 (expressing WT and Q311E MB21D2) in response to low dosage (2.5µM) of RAS inhibitor. MTT assay was used to determine cell viability. Statistical significance (EV control vs WT or Q311E): *, $p < 0.05$; **, $p < 0.01$; ***, $p < 0.001$. (WT vs Q311E): (WT vs Q311E): $^{+}$, $p < 0.05$; $^{++}$, $p < 0.01$; $^{+++}$, $p < 0.001$; ns: not significant.

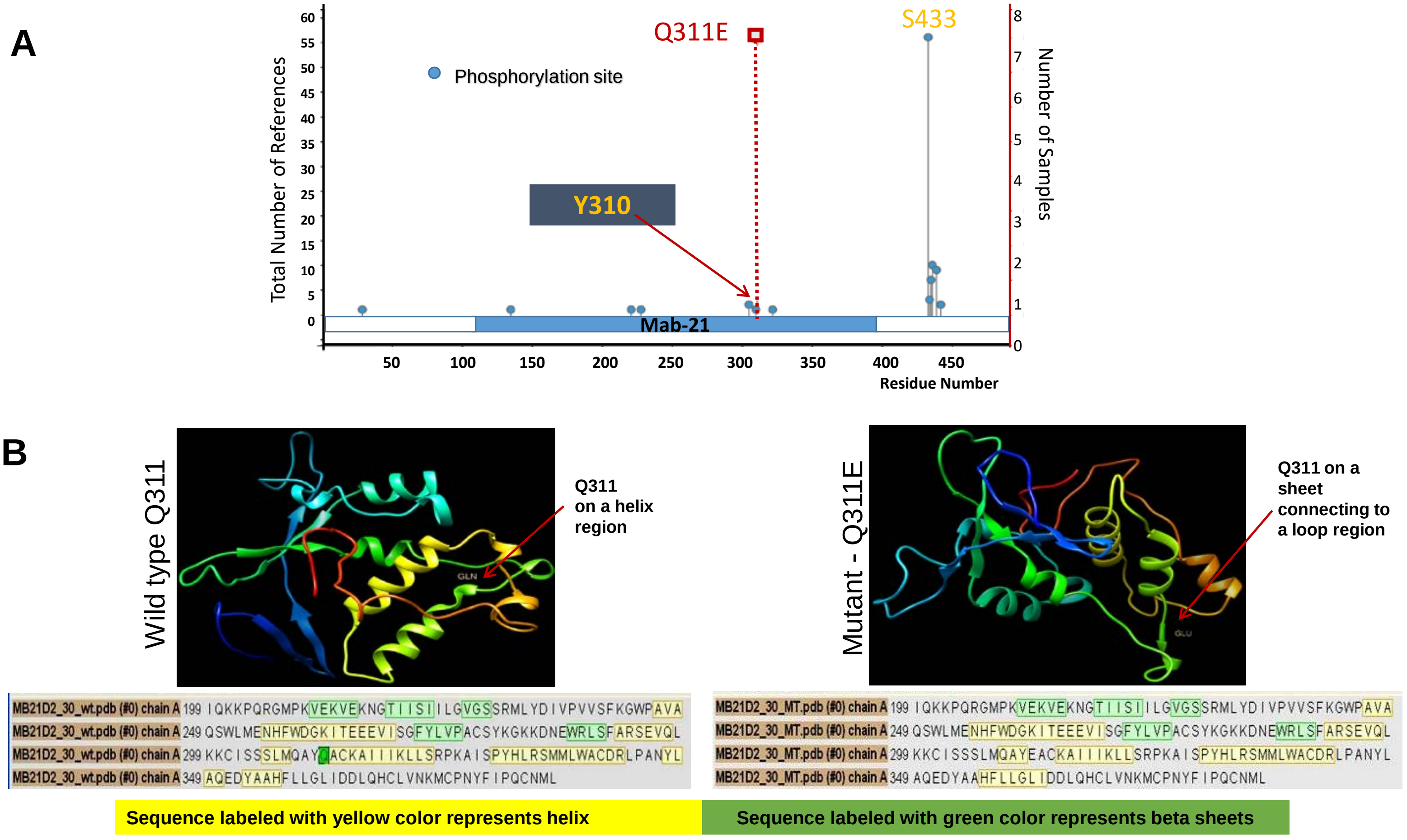

Figure S10. Mutation profile and structural predication of MB21D2 and its Q311E form**. A**. Phosphorylation and mutation site on MB21D2 based on PhosphoSitePlus (https://www.phosphosite.org). **B**. Predicted structure of MB21D2 model and its mutant type based on Swiss Prot. Database (https://swissmodel.expasy.org/).

A

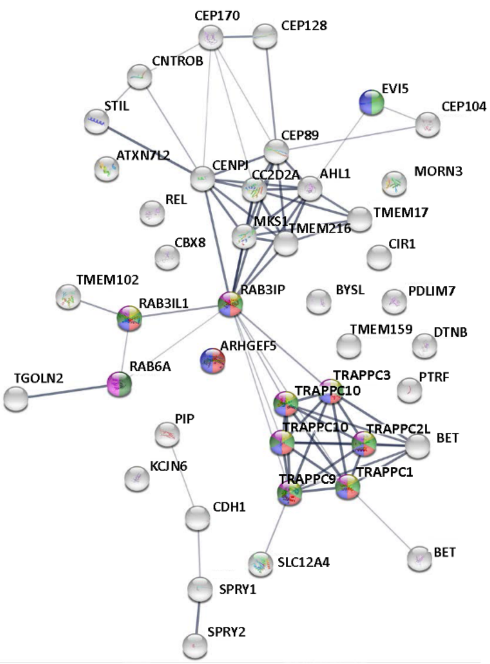

MOLECULAR FUNCTION / REACTOME PATHWAYS			
**GO-TERM**	**DESCRIPTION**	**COUNT IN GENE SET**	**FASLE DISCOVERY RATE**
GO:0017112	Rab guanyl-nucleotide exchange factor activity	8 of 45	3.54E-11
GO:0017137	Rab GTPase binding	9 of 176	1.67E-08
GO:0005088	Ras guanyl-nucleotide exchange factor activity	9 of 243	1.70E-07
GO:0017016	Ras GTPase binding	10 of 510	4.41E-06
HSA-8876198	RAB GEFs exchange GTP for GDP on RABs	9 of 86	7.22E-11
HSA-9007101	Rab regulation of trafficking	9 of 118	5.18E-10

B

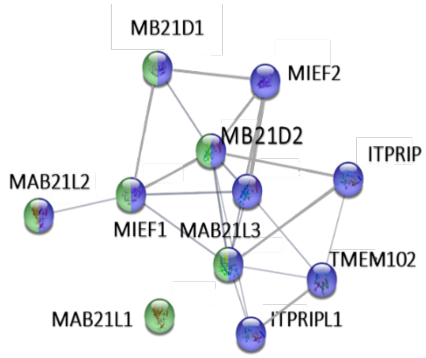

REFERENCE PUBLICATION			
**REFERENCE**	**YEAR(TITLE)**	**COUNT IN GENE SET**	**FASLE DISCOVERY RATE**
PMID:19833706	(2009) Comprehensive classification of nucleotidyltransferase fold proteins: identification of novel families and their representatives in human.	9 of 64	2.63E-18
PMID:27271801	(2016) Structural and biochemical characterization of the cell fate identification of novel families and their representatives in human.	6 of 11	5.25E-14

Figure S11. Known and predicted interaction of MB21D2. **A.** Interaction generated using STRING for all experimentally validated interactors of MB21D2 (by Affinity Capture Mass Spectrometry and/or Two Hybrid Assay) based on Bio-Grid database. **B.** Network of all known human Mab-domain containing genes generated using STRING database.

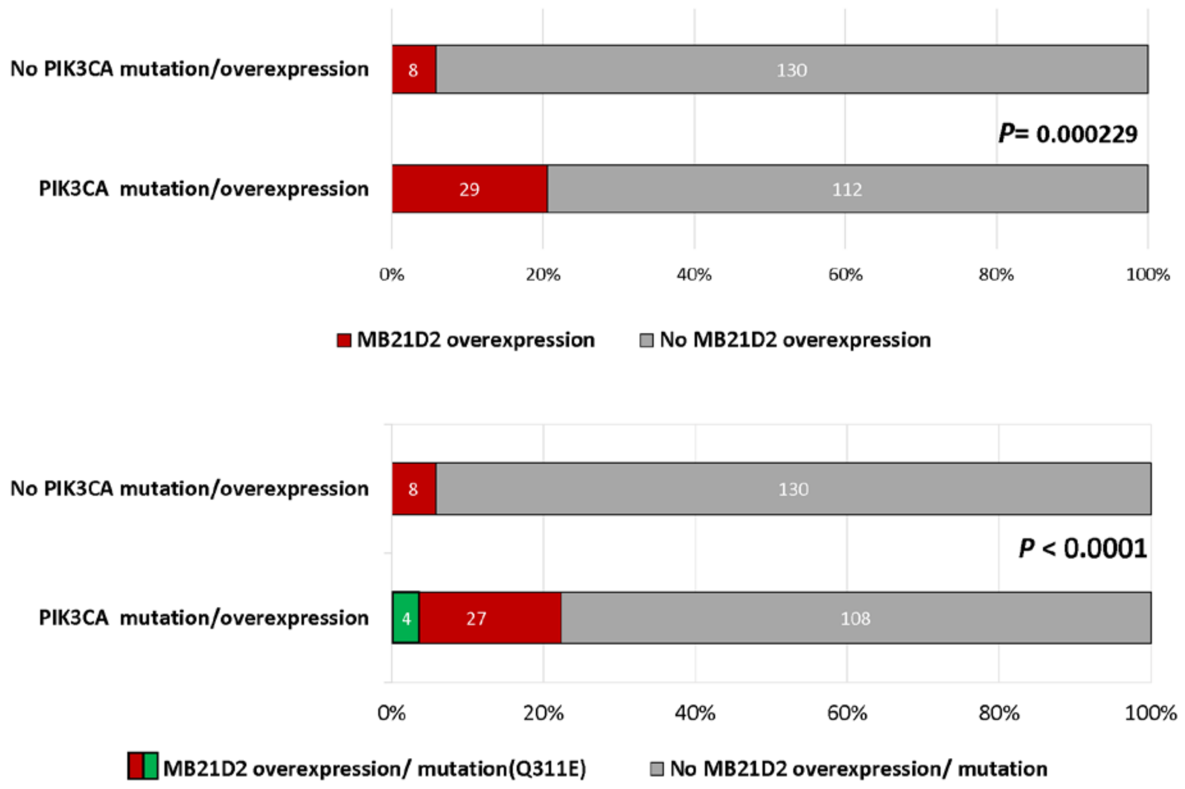

Figure S12. Association between MB21D2 overexpression and Q311E mutation with PIK3CA overexpression and mutation from actual patient sequencing data. **A.** Association between PIK3CA overexpression/mutation with MB21D2 mutation/overexpression. **B**. PIK3CA overexpression and mutation with MB21D2 overexpression plus Q311E mutation. Data were taken from TCGA (https://www.cbioportal.org/).

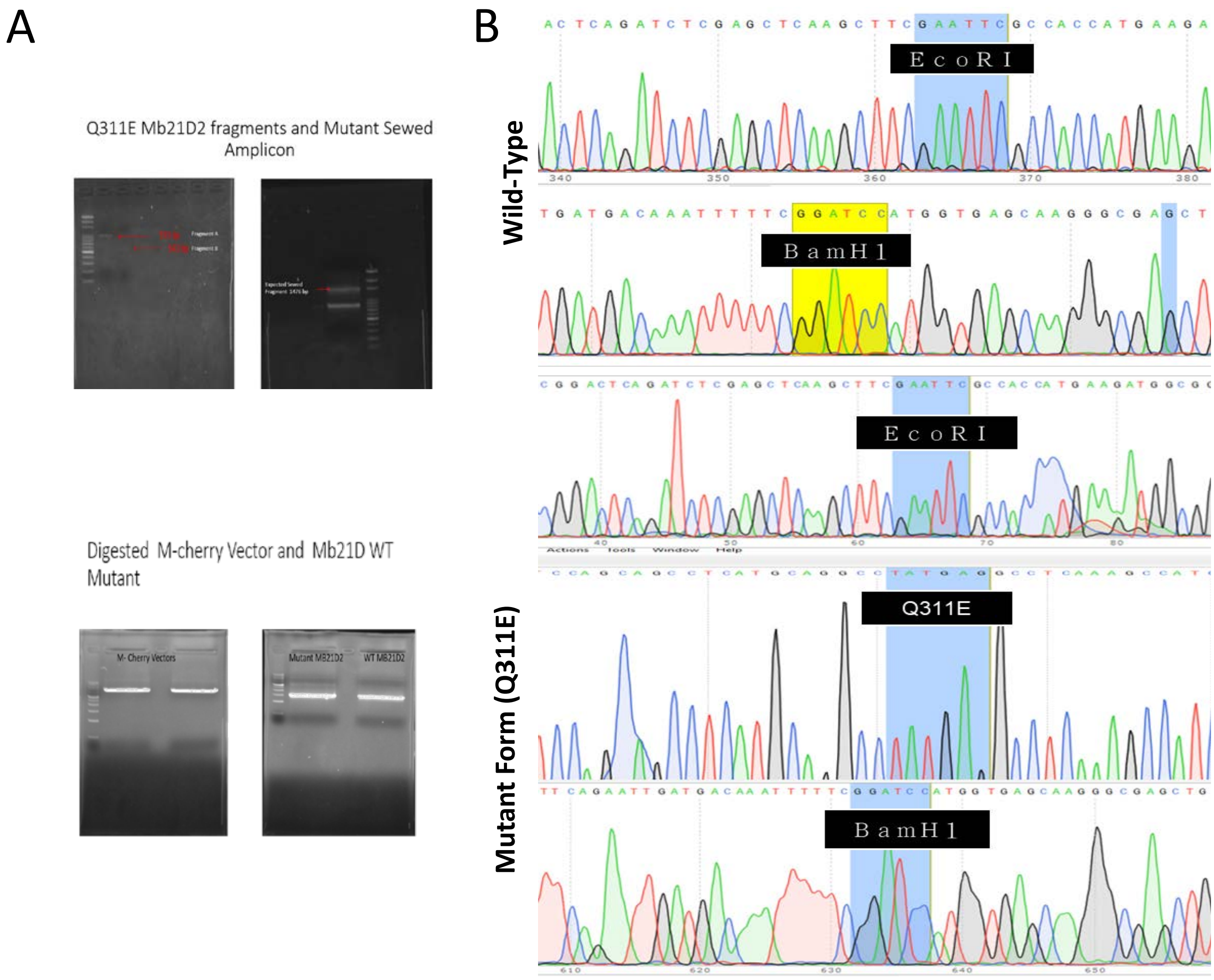

Figure S13. Cloning of wild type MB21D2 and Q311E form. **A.** Gel images of cloning and mutagenesis by overlap extension PCR, Vector and Insert on gel images. **B.** Sequences of WT and Q311E showing in-frame reading within the vector (Restriction sites EcoRI and BamHI).
