# Supplementary material for: Overexpression of wild type or a Q311E mutant MB21D2 promotes a pro‐oncogenic phenotype in HNSCC
Source: Mol Oncol. 2020 Oct 15;14(12):3065–82. doi: 10.1002/1878-0261.12806 (PMC7718949; doi:10.1002/1878-0261.12806)
Supplement: Supplementary file 2 — Table S1. Mutation rates of genes (312), annotated as cadherin binding (from UniProt database) based on 1490 patients, in four squamous cell carcinoma, namely, cervical squamous carcinoma (CESC), and esophageal squamous cell carcinoma (ESCA), lung squamous cell carcinoma (LUSC), and head and neck squamous cell carcinoma (HNSCC) obtained from TCGA sequencing databank. Table S2. Cadherin Binding Genes with ≥ 2% mutation rate in 1490 patients in four squamous cell carcinoma namely cervical squamous carcinoma (CESC), esophageal squamous cell carcinoma (ESCA), lung squamous cell carcinoma (LUSC), and head and neck squamous cell carcinoma (HNSCC) collected from TCGA sequencing databank. Table S3. Reactome and Genes Enriched by MB21D2 overexpression in clinical sample from TCGA sequencing data. Table S4. Positively correlated pathway/signature enriched by MB21D2(Wild‐type and Q311E) in stable cell clones based on transcriptome sequencing. Table S5. Co‐occurrence of MB21D2 with known PIK pathways regulators in HNSCC based on TCGA sequencing data. Table S6. List of Primers and antibody dilution used in the study. [file MOL2-14-3065-s002.pdf]

Supplementary Tables

Table S1. Mutation rates of genes (312), annotated as cadherin binding (from UniProt database) based on 1490 patients, in four squamous cell carcinoma, namely, cervical squamous carcinoma (CESC), and esophageal squamous cell carcinoma (ESCA), lung squamous cell carcinoma (LUSC), and head and neck squamous cell carcinoma (HNSCC) obtained from TCGA sequencing databank.

Gene	Rate	Gene	Rate	Gene	Rate	Gene	Rate	Gene	Rate	Gene	Rate	Gene	Rate	Gene	Rate
ABCF3	0.9%	KLC2	0.8%	CTNNA2	6%	RANGAP1	0.7%	CDH1	1.3%	PCBP1	0.5%	ERC1	2.1%	STK24	0.3%
ABI1	0.8%	KRT18	0.4%	CTNNA3	4%	RARS	0.6%	CDH10	7%	PCMT1	0.2%	ESYT2	1%	STK38	0.6%
ADD1	1.4%	KTN1	2.6%	CTNNAL1	0.9%	RDX	0.7%	CDH11	4%	PDLIM1	0.3%	EVPL	2.1%	STX5	0.8%
AFDN	2.9%	LAD1	0.6%	CTNNB1	1.3%	RPL14	0.1%	CDH12	3%	PDLIM5	0.8%	EXOC3	0.9%	STXBP6	0.2%
AHNAK	8%	LARP1	1.2%	CTNND1	2.1%	RPL15	0.1%	CDH13	1.7%	PDXDC1	0.7%	EZR	0.7%	SWAP70	0.7%
AHSA1	0.3%	LASP1	0.2%	CTNND2	5%	RPL23A	0%	CDH15	1.1%	PFKP	1.1%	F11R	0.4%	TACSTD2	0.2%
ALDOA	0.4%	LDHA	0.1%	CTTN	2.0%	RPL24	0.1%	CDH17	2.2%	PFN1	0.1%	FAM129B	0.8%	TAGLN2	0.1%
ANK3	5%	LIMA1	0.9%	DAB2IP	0.8%	RPL29	0.2%	CDH18	4%	PHLDB2	2.0%	FASN	3.0%	TBC1D10A	1%
ANLN	1.6%	LRRC59	0.2%	DBN1	0.9%	RPL34	0.1%	CDH19	2.9%	PI4KA	2.3%	FLNA	5%	TBC1D2	0.1%
ANXA1	0.2%	LRRFIP1	1%	DBNL	0.5%	RPL6	0.8%	CDH2	2.1%	PICALM	0.9%	FLNB	2.1%	TES	0.3%
ANXA2	0.1%	LYPLA2	0.1%	DDX3X	1.7%	RPL7A	0.5%	CDH20	3.0%	PKM	0.9%	FMNL2	0.8%	TJP1	2.9%
ARFIP2	0.3%	MACF1	8%	DDX6	0.5%	RPS2	0.2%	CDH22	0.7%	PKN2	1.1%	FNBP1L	0.1%	TJP2	2.0%
ARGLU1	0.4%	MAPRE1	0.4%	DHX29	0.9%	RPS26	0.1%	CDH24	1.2%	PKP1	0.5%	FSCN1	0.4%	TLN1	4%
ARHGAP1	0.5%	MARK2	1.1%	DIAPH3	2.5%	RSL1D1	0.5%	CDH26	1.2%	PKP2	1.5%	FXYD5	0.7%	TMOD3	0.2%
ARHGAP18	0.8%	MB21D2	2.7%	DLG1	2.0%	RTN4	1.4%	CDH3	1.3%	PKP3	1.3%	GAPVD1	1.5%	TMPO	0.9%
ARHGEF16	0.6%	MICALL1	0.9%	DNAJB1	0.8%	RUVBL1	0.7%	CDH4	2.6%	PKP4	2.8%	GCN1	2.1%	TNKS1BP1	3%
ARVCF	1%	MMP24	0.5%	DOCK9	2.0%	S100A11	0.2%	CDH5	2.5%	PLCB3	2.1%	GIGYF2	2.7%	TRIM25	0.4%
ASAP1	2.7%	MPP7	1.2%	EEF1D	0.2%	S100P	0.1%	CDH6	2.6%	PLEC	8%	GIPC1	0.4%	TRIM29	0.2%
ATIC	0.7%	MPRIP	1%	EEF1G	0.7%	SCRIB	2.8%	CDH7	4%	PLIN3	0.3%	GLOD4	0.1%	TRPC4	2.5%
ATXN2L	1.7%	MRE11	0.5%	EEF2	1.1%	SCYL1	0.6%	CDH8	4%	PPFIBP1	1.1%	GOLGA2	0.6%	TWF1	0.1%
BAG3	0.8%	MRTFB	1.1%	EFHD2	0.1%	SERBP1	0.8%	CDH9	6%	PPL	2.8%	GOLGA3	3%	TWF2	0.6%
BAIAP2	0.4%	MYH9	5%	EGFR	2.8%	SFN	1%	CDHR3	1.3%	PPME1	0.3%	GPRC5A	0.4%	TXNDC9	0.3%
BAIAP2L1	0.8%	MYO1B	1.5%	EHD1	0.9%	SH3GL1	0.7%	CDK5R1	0.2%	PPP1CA	0.5%	H1FX	0.3%	UBAP2	1.6%
BSG	0.4%	NCK1	0.8%	EHD4	0.6%	SH3GLB1	0.4%	CEMIP2	2.6%	PPP1R13L	2.2%	HCFC1	1.8%	UBFD1	0.3%
BZW1	0.4%	NDRG1	0.7%	EIF2A	0.6%	SH3GLB2	0.3%	CGN	2.2%	PRDX1	0.2%	HDLBP	1.6%	UNC45A	1.2%
BZW2	0.3%	NOP56	0.7%	EIF2S3	0.1%	SHTN1	0.6%	CHMP2B	0.5%	PRDX6	0.4%	HIST1H3A	0.3%	USO1	0.5%
CALD1	0.9%	NOTCH3	5%	EIF3E	0.8%	SLC3A2	0.9%	CHMP4B	0.3%	PROM1	1.2%	HNRNPK	0.4%	USP8	2.2%
CAPG	0.4%	NUDC	0.4%	EIF4G2	1.1%	SLC9A3R2	0.5%	CHMP5	0.3%	PSEN1	0.7%	HSP90AB1	1.2%	VAPA	0.2%
CAPZA1	0.5%	NUMB	0.6%	EIF4H	0.1%	SLK	0.8%	CIP2A	2.2%	PSMB6	0.2%	HSPA1A	0%	VAPB	0.1%
CAPZB	0.5%	OLA1	0.5%	EIF5	0.9%	SND1	0.9%	CKAP5	2%	PTPN1	0.5%	HSPA5	0.5	VASN	0.2%
CAST	0.6%	OLFM4	1.5%	EMD	0.3%	SNX1	0.6%	CLIC1	0.3%	PTPRM	3%	HSPA8	2%	VASP	0.4%
CBL	0.7%	P2RX4	0.5%	ENO1	0.5%	SNX2	0.2%	CLINT1	0.7%	PTPRT	5%	IDH1	0.6%	VCL	1.1%
CC2D1A	1.8%	PACSIN2	0.9%	EPCAM	0.5%	SNX5	0.4%	CNN2	0.2%	PUF60	0.7%	IQGAP1	2.8%	WASF2	0.7%
CCNB2	0.1%	PAICS	0.2%	EPHA2	4%	SNX9	0.3%	CNN3	0.3%	RAB10	0.2%	IST1	0.2%	YKT6	0.4%
CCS	0.5%	PAK2	1.2%	EPN2	0.6%	SPTAN1	4%	COBLL1	2.5%	RAB11B	0.3%	ITGA6	1.2%	YWHAB	0.3%
CCT8	0.3%	PAK4	0.5%	EPS15	2.2%	SPTBN1	4%	CORO1B	0.4%	RAB1A	0.2%	ITGAE	2.3%	YWHAE	0.4%
CD2AP	0.6%	PAK6	1%	EPS15L1	1.1%	SPTBN2	3%	CRKL	0.4%	RACK1	0.3%	ITGB1	2.1%	YWHAZ	0.6%
CD46	0.4%	PARK7	0.2%	EPS8L1	1.3%	SRC	0.1%	CSNK1D	0.7%	RAN	0.2%	JUP	1.5%	ZC3H15	0.7%
CDC42EP1	0.8%	PARVA	0.2%	EPS8L2	0.6%	STAT1	1.2%	CTNNA1	1.3%	RANBP1	0.2%	KIF5B	1.2%	ZC3HAV1	1%

Table S2. Cadherin Binding Genes with ≥ 2% mutation rate in 1490 patients in four squamous cell carcinoma namely cervical squamous carcinoma (CESC), esophageal squamous cell carcinoma (ESCA), lung squamous cell carcinoma (LUSC),and head and neck squamous cell carcinoma (HNSCC) collected from TCGA sequencing databank.

Gene Name	Mutation Rate	Recurrent Mutation Rate	Cytogenetic Band	Function (Based On Uniprot Annotation)
MACF1	8%	1.6	1p34.3	Plays a role in cross-linking actin to other cytoskeletal proteins and also binds to microtubules
PLEC	8%	0.0	8q24.3	Interlinks intermediate filaments with microtubules and microfilaments
AHNAK	8%	0.0	11q12.3	May be required for neuronal cell differentiation.
CTNNA2	6%	0.0	2p12	May function as a linker between cadherin adhesion receptors and the cytoskeleton to regulate cell-cell adhesion and differentiation in the nervous system (By similarity). Required for proper regulation of cortical neuronal migration and neurite growth (PubMed:30013181). It acts as negative regulator of Arp2/3 complex activity and Arp2/3-mediated actin polymerization (PubMed:30013181). It thereby suppresses excessive actin branching which would impair neurite growth and stability (PubMed:30013181). Regulates morphological plasticity of synapses and cerebellar and hippocampal lamination during development. Functions in the control of startle modulation (By similarity).By similarity
CDH10	7%	0.0	5p14.2-p14.1	T2 cadherin,transmembrane glycoprotein,component of adherens junction
PTPRT	5%	0.0	20q12-q13.11	May be involved in both signal transduction and cellular adhesion in the CNS.
ANK3	5%	2.4	10q21.2	Required for costamere localization of DMD and betaDAG1 . Membrane-cytoskeleton linker.
CTNND2	5%	0.0	5p15.2	Has a critical role in neuronal development, particularly in the formation and/or maintenance of dendritic spines and synapses (PubMed:25807484). Involved in the regulation of Wnt signaling (PubMed:25807484). It probably acts on beta-catenin turnover, facilitating beta-catenin interaction with GSK3B, phosphorylation, ubiquitination and degradation (By similarity). Functions as a transcriptional activator when bound to ZBTB33 (By similarity). May be involved in neuronal cell adhesion and tissue morphogenesis and integrity by regulating adhesion molecules.
CDH18	4%	4.8	5p14.3	R-cadherin, transmembrane glycoprotein cell adhesion molecule,
CDH9	6%	0.0	5p14.1	homophilic interaction
NOTCH3	5%	3.6	19p13.12	Receptor for membrane-bound ligands Jagged1, Jagged2 and Delta1 to regulate cell-fate determination

MYH9	5%	6.0	22q12.3	Contractile protein moving towards the "plus" end of actin track,heavy polypeptide 9,non muscle
FLNA	5%	4.0	Xq28	Promotes orthogonal branching of actin filaments and links actin filaments to membrane glycoproteins. Anchors various transmembrane proteins to the actin cytoskeleton and serves as a scaffold for a wide range of cytoplasmic signaling proteins. Interaction with FLNA may allow neuroblast migration from the ventricular zone into the cortical plate. Tethers cell surface-localized furin, modulates its rate of internalization and directs its intracellular trafficking (By similarity). Involved in ciliogenesis. Plays a role in cell-cell contacts and adherens junctions during the development of blood vessels, heart and brain organs. Plays a role in platelets morphology through interaction with SYK that regulates ITAM- and ITAM-like-containing receptor signaling, resulting in by platelet cytoskeleton organization maintenance (By similarity).By similarity1 Publication
CDH8	4%	0.0	16q21	Transmembrane glycoprotein component of adherens junction
CDH7	4%	0.0	18q22.1	BH-cadherin chicken cadherin 7-like,transmembrane glycoprotein component of adherens junction
PTPRM	3%	0.0	18p11.23	Involved in cell-cell adhesion through homophilic interactions.
GOLGA3	3%	0.0	12q24.33	Golgi auto-antigen; probably involved in maintaining Golgi structure.
CDH12	3%	4.0	5p14.3	homophilic interaction
CDH11	4%	0.0	16q21	Interact with themselves in a homophilic manner in connecting cells
TLN1	4%	4.7	9p13.3	Probably involved in connections of major cytoskeletal structures to the plasma membrane.
TJP1	2.9%	0.0	15q13.1	required for stable association with the tight junction
SPTBN1	4%	0.0	2p16.2	Interacts with calmodulin in a calcium-dependent manner
SPTAN1	4%	4.4	9q34.11	Fodrin, which seems to be involved in secretion, interacts with calmodulin in a calcium-dependent manner and is thus candidate for the calcium-dependent movement of the cytoskeleton at the membrane
SPTBN2	3%	0.0	11q13.2	plays an important role in neuronal membrane skeleton
TRPC4	2.5%	5.7	13q13.3	Form a receptor-activated non-selective calcium permeant cation channel. Acts as a cell-cell contact-dependent endothelial calcium entry channel.
SCRIB	2.8%	0.0	8q24.3	May play a role in exocytosis and in the targeting synaptic vesicles to synapses.
TNKS1BP1	3%	5.4	11q12.1	cadherin binding, protein binding complex,ankyrin repeat binding
EPHA2	4%	8.0	1p36.13	Receptor tyrosine kinase which binds promiscuously membrane-bound ephrin-A family ligands

CTNNA3	4%	0.0	10q21.3	May be involved in formation of stretch-resistant cell-cell adhesion complexes.
EGFR	2.8%	5.4	7p11.2	Receptor tyrosine kinase binding ligands of the EGF family and activating several signaling cascades to convert extracellular cues into appropriate cellular responses
FLNB	2.1%	0.0	3p14.3	Connects cell membrane constituents to the actin cytoskeleton. May promote orthogonal branching of actin filaments and links actin filaments to membrane glycoproteins. Anchors various transmembrane proteins to the actin cytoskeleton. Interaction with FLNA may allow neuroblast migration from the ventricular zone into the cortical plate. Various interactions and localizations of isoforms affect myotube morphology and myogenesis. Isoform 6 accelerates muscle differentiation in vitro
CDH2	2.1%	9.3	18q12.1	Calcium-dependent cell adhesion protein; preferentially mediates homotypic cell-cell adhesion by dimerization with a CDH2 chain from another cell. Cadherins may thus contribute to the sorting of heterogeneous cell types. Acts as a regulator of neural stem cells quiescence by mediating anchorage of neural stem cells to ependymocytes in the adult subependymal zone: upon cleavage by MMP24, CDH2-mediated anchorage is affected, leading to modulate neural stem cell quiescence. CDH2 may be involved in neuronal recognition mechanism. In hippocampal neurons, may regulate dendritic spine density
FASN	3%	9.1	17q25.3	Fatty acid synthetase catalyzes the formation of long-chain fatty acids from acetyl-CoA, malonyl-CoA and NADPH. This multifunctional protein has 7 catalytic activities as an acyl carrier protein
GCN1	2.1%	0.0	12q24.23	Acts as a positive activator of the EIF2AK4/GCN2 protein kinase activity in response to amino acid starvation. Forms a complex with EIF2AK4/GCN2 on translating ribosomes; during this process, GCN1 seems to act as a chaperone to facilitate delivery of uncharged tRNAs that enter the A site of ribosomes to the tRNA-binding domain of EIF2AK4/GCN2, and hence stimulating EIF2AK4/GCN2 kinase activity. Participates in the repression of global protein synthesis and in gene-specific mRNA translation activation, such as the transcriptional activator ATF4, by promoting the EIF2AK4/GCN2-mediated phosphorylation of eukaryotic translation initiation factor 2 (eIF-2-alpha/EIF2S1) on 'Ser-52', and hence allowing ATF4-mediated reprogramming of amino acid biosynthetic gene expression to alleviate nutrient depletion
CEMIP2	2.6%	7.8	9q21.13	Cell surface hyaluronidase that mediates the initial cleavage of extracellular hyaluronan
DOCK9	2.0%	5.5	13q32.3	Guanine nucleotide-exchange factor (GEF) that activates CDC42 by exchanging bound GDP for free GTP. Overexpression induces filopodia formation.

MB21D2	2.7%	35.5	3q29	cadherin binding, protein binding complex
CDH19	2.9%	5.8	18q22.1	homophilic interaction
CDH20	3%	0.0	18q21.33	Cadherins are calcium-dependent cell adhesion proteins. They preferentially interact with themselves in a homophilic manner in connecting cells; cadherins may thus contribute to the sorting of heterogeneous cell types.
IQGAP1	2.8%	0.0	15q26.1	dynamics and assembly of the actin cytoskeleton
AFDN	2.9%	0.0	6q27	May play a role in the organization of homotypic, interneuronal and heterotypic cell-cell adherens junctions
PPL	2.8%	0.0	16p13.3	Component of the cornified envelope of keratinocytes.
PKP4	2.8%	6.6	2q24.1	Plays a role as a regulator of Rho activity during cytokinesis. May play a role in junctional plaques
ASAP1	2.7%	6.8	8q24.21-q24.22	Possesses phosphatidylinositol 4,5-bisphosphate-dependent GTPase-activating protein activity for ARF1 (ADP ribosylation factor 1) and ARF5 and a lesser activity towards ARF6. Plays a role in ciliogenesis.
GIGYF2	2.7%	7.6	2q37.1	Key component of the 4EHP-GYF2 complex, a multiprotein complex that acts as a repressor of translation initiation (PubMed:22751931). In 4EHP-GYF2 the complex, acts as a factor that bridges EIF4E2 to ZFP36/TTP, linking translation repression with mRNA decay (By similarity). May act cooperatively with GRB10 to regulate tyrosine kinase receptor signaling, including IGF1 and insulin receptors (PubMed:12771153)
CDH4	2.6%	6.6	20q13.33	Cadherins are calcium-dependent cell adhesion proteins. They preferentially interact with themselves in a homophilic manner in connecting cells; cadherins may thus contribute to the sorting of heterogeneous cell types. May play an important role in retinal development.
CDH6	2.6%	11.0	5p13.3	K-cadherin,transmembrane glycoprotein component of adherens junction
KTN1	2.6%	0.0	14q22.1	Receptor for kinesin thus involved in kinesin-driven vesicle motility. Accumulates in integrin-based adhesion complexes (IAC) upon integrin aggregation by fibronectin.
EVPL	2.1%	0.0	17q25.1	Component of the cornified envelope of keratinocytes. May link the cornified envelope to desmosomes and intermediate filaments.

CDH5	2.5%	8.0	16q21	Cadherins are calcium-dependent cell adhesion proteins (By similarity). They preferentially interact with themselves in a homophilic manner in connecting cells; cadherins may thus contribute to the sorting of heterogeneous cell types (PubMed:21269602). This cadherin may play a important role in endothelial cell biology through control of the cohesion and organization of the intercellular junctions (By similarity). It associates with alpha-catenin forming a link to the cytoskeleton (PubMed:10861224). Acts in concert with KRIT1 and MPP5 to establish and maintain correct endothelial cell polarity and vascular lumen (By similarity). These effects are mediated by recruitment and activation of the Par polarity complex and RAP1B (PubMed:20332120). Required for activation of PRKCZ and for the localization of phosphorylated PRKCZ, PARD3, TIAM1 and RAP1B to the cell junction (PubMed:20332120)
USP8	2.2%	0.0	15q21.2	required for endosomal localization, CHMP1B-binding, maintenance of ESCRT-0 stability and EGFR degradation.
COBLL1	2.5%	12.0	2q24.3	actin monomer binding
CKAP5	2.0%	7.6	11p11.2	Binds to the plus end of microtubules and regulates microtubule dynamics and microtubule organization. Acts as processive microtubule polymerase. Promotes cytoplasmic microtubule nucleation and elongation. Plays a major role in organizing spindle poles. In spindle formation protects kinetochore microtubules from depolymerization by KIF2C and has an essential role in centrosomal microtubule assembly independently of KIF2C activity. Contributes to centrosome integrity. Acts as component of the TACC3/ch-TOG/clathrin complex proposed to contribute to stabilization of kinetochore fibers of the mitotic spindle by acting as inter-microtubule bridge. The TACC3/ch-TOG/clathrin complex is required for the maintenance of kinetochore fiber tension (PubMed:23532825). Enhances the strength of NDC80 complex-mediated kinetochore-tip microtubule attachments (PubMed:27156448).
DIAPH3	2.5%	0.0	13q21.2	Actin nucleation and elongation factor
ITGAE	2.3%	8.3	17p13.2	Integrin alpha-E/beta-7 is a receptor for E-cadherin. It mediates adhesion of intra-epithelial T-lymphocytes to epithelial cell monolayers.
PI4KA	2.3%	9.5	22q11.21	Acts on phosphatidylinositol (PtdIns) in the first committed step in the production of the second messenger inositol-1,4,5,-trisphosphate.

CDH17	2.2%	0.0	8q22.1	Cadherins are calcium-dependent cell adhesion proteins. They preferentially interact with themselves in a homophilic manner in connecting cells; cadherins may thus contribute to the sorting of heterogeneous cell types. LI-cadherin may have a role in the morphological organization of liver and intestine. Involved in intestinal peptide transport
CIP2A	2.2%	0.0	3q13.13	Oncoprotein that inhibits PP2A and stabilizes MYC in human malignancies. Promotes anchorage-independent cell growth and tumor formation
EPS15	2.2%	0.0	1p32.3	Involved in cell growth regulation. May be involved in the regulation of mitogenic signals and control of cell proliferation. Involved in the internalization of ligand-inducible receptors of the receptor tyrosine kinase (RTK) type, in particular EGFR. Plays a role in the assembly of clathrin-coated pits (CCPs). Acts as a clathrin adapter required for post-Golgi trafficking. Seems to be involved in CCPs maturation including invagination or budding. Involved in endocytosis of integrin beta-1 (ITGB1) and transferrin receptor (TFR); internalization of ITGB1 as DAB2-dependent cargo but not TFR seems to require association with DAB2.
CGN	2.2%	8.0	1q21.3	Probably plays a role in the formation and regulation of the tight junction (TJ) paracellular permeability barrier.
ITGB1	2.1%	8.3	10p11.22	receptors for collagen.
PPP1R13L	2.2%	8.0	19q13.32	Regulator that plays a central role in regulation of apoptosis and transcription via its interaction with NF-kappa-B and p53/TP53 proteins. Blocks transcription of HIV-1 virus by inhibiting the action of both NF-kappa-B and SP1. Also inhibits p53/TP53 function, possibly by preventing the association between p53/TP53 and ASPP1 or ASPP2, and therefore suppressing the subsequent activation of apoptosis (PubMed:12524540)
PLCB3	2.1%	0.0	11q13.1	he production of the second messenger molecules diacylglycerol (DAG) and inositol 1,4,5-trisphosphate (IP3) is mediated by activated phosphatidylinositol-specific phospholipase C enzymes.
ERC1	2.1%	0.0	12p13.33	Regulatory subunit of the IKK complex. Probably recruits IkappaBalpha/NFKBIA to the complex. May be involved in the organization of the cytomatrix at the nerve terminals active zone (CAZ) which regulates neurotransmitter release. May be involved in vesicle trafficking at the CAZ. May be involved in Rab-6 regulated endosomes to Golgi transport.

CTNND1	2.1%	0.0	11q12.1	Binds to and inhibits the transcriptional repressor ZBTB33, which may lead to activation of target genes of the Wnt signaling pathway (By similarity). Associates with and regulates the cell adhesion properties of both C-, E- and N-cadherins, being critical for their surface stability. Implicated both in cell transformation by SRC and in ligand-induced receptor signaling through the EGF, PDGF, CSF-1 and ERBB2 receptors. Promotes GLIS2 C-terminal cleavage.By similarity2 Publications
HSPA8	2.0%	0.0	11q24.1	Protein stability, degradation and processing
PHLDB2	20%	0.0		Seems to be involved in the assembly of the postsynaptic apparatus. May play a role in acetyl-choline receptor (AChR) aggregation in the postsynaptic membrane
DLG1	2.0%	0.0	3q29	During embryonic development, some isoforms are essential for proper neuronal differentiation and organization. Required for cell polarity; maintenance of apicobasal polarity. Plays a critical role at septate junctions in cellular growth control during larval development. The presence of a guanylate kinase domain suggests involvement in cellular adhesion as well as signal transduction to control cellular proliferation.3 Publications
TJP2	2%	10.0	9q21.11	Plays a role in tight junctions and adherens junctions.
CTTN	2%	9.5	11q13.3	Contributes to the organization of the actin cytoskeleton and cell shape

Table S3. Reactome and Genes Enriched by MB21D2 overexpression in clinical sample from TCGA sequencing data.

REACTOME_ION_ TRANSPORT_BY_P_TYPE_ATPASES	Gene	RANK IN GENE LIST	RANK METRIC SCORE	RUNNING ES	CORE ENRICHMENT	Co-occurrence p value
p value 0.015151516	ATP11B	63	0.3740633	0.25793475	Yes	<0.001
	ATP8B1	176	0.2746827	0.44413158	Yes	<0.001
	ATP1B3	988	0.1782112	0.5288791	Yes	<0.001
	ATP8A1	1960	0.1404395	0.57945716	Yes	<0.001
REACTOME_ION_CHANNEL_ TRANSPORT						
p value 0.016438356	ATP11B	63	0.3740633	0.10969835	Yes	<0.001
	ATP8B1	176	0.2746827	0.1870389	Yes	<0.001
	GABRR2	621	0.2032437	0.22661264	Yes	<0.001
	ATP1B3	988	0.1782112	0.26245195	Yes	<0.001
	GLRA3	1752	0.1471964	0.26953486	Yes	<0.001
	GLRB	1765	0.1466774	0.31317064	Yes	<0.001
	GABRA5	1791	0.1457825	0.35590118	Yes	<0.001
	GABRR1	1831	0.144558	0.3975782	Yes	<0.001
	ATP8A1	1960	0.1404395	0.43366313	Yes	<0.001
	GABRG3	2379	0.1294157	0.4522491	Yes	<0.001
	GABRB3	2714	0.1216289	0.47259337	Yes	<0.001
	GLRA1	3083	0.1135806	0.48884916	Yes	<0.001
REACTOME_YAP1_AND_WWTR1_TAZ_STIMULATED_GENE_EXPRESSION						
p value 0.01910828	TBL1XR1	4	0.5476644	0.31966442	Yes	<0.001
	WWTR1	172	0.2759134	0.47265628	Yes	<0.001
	NCOA1	1027	0.1759754	0.5337384	Yes	<0.001
	CREBBP	1415	0.1585544	0.6074464	Yes	<0.001
REACTOME_TRANSPORT_OF_VITAMINS_NUCLEOSIDES_AND_RELATED_MOLECULES						
p value0.020997375	SLC33A1	41	0.4158438	0.20096748	Yes	<0.001
	SLCO3A1	188	0.2723366	0.32676148	Yes	<0.001
	SLC35A3	1009	0.1773396	0.37326685	Yes	<0.001
	SLC29A4	1253	0.1660192	0.44243023	Yes	<0.001
	SLC29A2	1975	0.1401663	0.47562718	Yes	<0.001
REACTOME_TIGHT_JUNCTION_INTERACTIONS						
	PRKCI	28	0.438095	0.14692457	Yes	<0.001

p value 0.032994922	PARD3	357	0.2301214	0.20879805	Yes	<0.001
	CLDN15	480	0.215816	0.27589127	Yes	<0.001
	CLDN20	809	0.1888257	0.3237864	Yes	<0.001
	F11R	1115	0.1714551	0.36692515	Yes	<0.001
	CLDN11	1408	0.1587848	0.40641007	Yes	<0.001
	CLDN7	1462	0.1570005	0.4569649	Yes	<0.001
	CLDN16	1926	0.1416105	0.48228398	Yes	<0.001
	CLDN8	2156	0.1348826	0.51675534	Yes	<0.001
REACTOME_SIGNALING_BY_FGFR_MUTANTS						
	PIK3CA	51	0.392307	0.11219714	Yes	<0.001
p value 0.041666668	CPSF6	316	0.2372674	0.16865835	Yes	<0.001
	FGFR2	636	0.2019517	0.21210691	Yes	<0.001
	CNTRL	1507	0.1551275	0.214936	Yes	<0.001
	ZMYM2	1530	0.1542103	0.25894368	Yes	<0.001
	TRIM24	1995	0.1394445	0.27703145	Yes	<0.001
	FGF9	2027	0.1385733	0.31602782	Yes	<0.001
	KRAS	3124	0.1127708	0.2954281	Yes	<0.001
	FGF4	3466	0.1064447	0.3098802	Yes	<0.001
	STAT5B	3540	0.1050864	0.33703396	Yes	<0.001
	FGF18	3955	0.0979348	0.3454303	Yes	<0.001
	FGFR3	4366	0.0905489	0.3518629	Yes	<0.001
	FGFR1OP2	4369	0.0905154	0.37822706	Yes	<0.001
	FGF3	4686	0.0852226	0.38769686	Yes	<0.001
	FGFR4	5228	0.0759079	0.38344648	Yes	<0.001
	FGF17	5650	0.0695618	0.38320592	Yes	<0.001
	FGF7	5707	0.068773	0.40057445	Yes	<0.001
	FGF1	5928	0.0658194	0.40906388	Yes	<0.001
	STAT5A	6279	0.0607332	0.40971252	Yes	<0.001
	FGF10	6439	0.0583864	0.41901034	Yes	<0.001
REACTOME_RORA_ACTIVATES_CIRCADIAN_EXPRESSION						
p value0.04338843	TBL1XR1	4	0.5476644	0.3385158	Yes	<0.001
	NCOA1	1027	0.1759754	0.39745045	Yes	<0.001
	CPT1A	1045	0.1748402	0.50475293	Yes	<0.001
	CREBBP	1415	0.1585544	0.5847965	Yes	<0.001
	RORA	2633	0.1233914	0.60168874	Yes	<0.001
	EP300	3428	0.1069847	0.6290874	Yes	<0.001

Table S4. Positively correlated pathway/signature enriched by MB21D2(WILD-TYPE AND Q311E) in stable cell clones based on transcriptome sequencing.

**Wild-Type MB21D2**								
**ID**	**setSize**	**Enrichment Score**	**NES**	**pvalue**	**p.adjust**	**rank_at_max**	**leading_edge**	**core_enrichment**
KRAS.50_UP.V1_UP	24	0.376239	1.47363	0.025	0.0773	989	tags=29%, list=6%, signal=27%	SNAP91/FAM155B/KIF5C/DOCK4/DYNC1I1/ETV1/GLDC
KRAS.KIDNEY_UP.V1_UP	71	0.49025	2.550943	0.038462	0.1093	989	tags=31%, list=6%, signal=29%	GRIA2/PLP1/PCDH9/SNAP91/SCN2A/CRYAB/NRN1/SOBP/MEF2C/RAPGEF4/GRIK2/ANK2/GPM6A/KIF5C/CPEB3/CPE/DDX6/NMNAT2/DYNC1I1/ETV1/SATB1/GLDC
BRCA1_DN.V1_UP	51	0.190468	0.890534	0.675	0.8752	1348	tags=20%, list=8%, signal=18%	TLX3/APBB1/PTPRS/MAP2/SP4/SIX1/CDKL5/OSR2/RCN3/HOXB7
CAHOY_NEURONAL	46	0.212533	0.997561	0.488372	0.6801	263	tags=7%, list=2%, signal=6%	EPHA7/SLC6A17/MEF2C
IL2_UP.V1_DN	103	0.188118	0.995541	0.454545	0.6609	696	tags=13%, list=4%, signal=12%	PBX1/ISL1/MAGI2/RORB/TEK/H2AFY2/PAK3/PRKG1/HOXA5/HDAC5/FZD2/MAP2K6/RASGRP3
KRAS.300_UP.V1_UP	69	0.224475	1.13792	0.2	0.3798	989	tags=16%, list=6%, signal=15%	SNAP91/SCN2A/FAM155B/KCNH2/KIF5C/DOCK4/DDX6/DYNC1I1/ETV1/SPRY2/GLDC
KRAS.50_UP.V1_DN	21	0.203421	0.781258	0.789474	0.9166	12	tags=5%, list=0%, signal=5%	SOX11
KRAS.600_UP.V1_UP	137	0.246929	1.446457	0.125	0.2611	989	tags=15%, list=6%, signal=15%	FOXG1/Cdhg/PCDH9/IGF2/SNAP91/SCN2A/FAM155B/ZEB1/REEP2/KCNH2/KIF5C/GALR2/DOCK4/NR4A3/DDX6/NMNAT2/DYNC1I1/ETV1/SPRY2/SATB1/GLDC
KRAS.AMP.LUNG_UP.V1_UP	44	0.256111	1.192299	0.121951	0.2576	1355	tags=20%, list=8%, signal=19%	NOVA1/FAM155B/EFNA3/SOX12/DOCK3/DYNC1I1/RAB30/SLC6A6/HSPA1L
KRAS.BREAST_UP.V1_DN	65	0.221291	1.078298	0.32	0.5418	562	tags=11%, list=3%, signal=10%	PBX1/PPM1E/SOBP/PTENP1/MAML3/HOXB5/COL5A2
KRAS.PROSTATE_UP.V1_UP	67	0.178156	0.887865	0.75	0.8913	656	tags=12%, list=4%, signal=12%	FAM155B/ZEB1/REEP2/TWIST1/NBEA/NR4A3/DNM1/ADRA1B
PTEN_DN.V1_UP	89	0.179683	0.956593	0.615385	0.8128	1063	tags=16%, list=6%, signal=15%	H2AFY2/HOXB2/HOXC8/LHX2/NR2F1/SSBP2/BDNF/NR4A3/TUSC3/KALRN/ZBTB10/GNAO1/PRKAR2B/SPOCK2
**Mutant(Q311E)**								

**ID**	**setSize**	**enrichment Score**	**NES**	**pvalue**	**p.adjust**	**rank_at _max**	**leading_edge**	**core_enrichment**
KRAS.50_UP.V1_UP	25	0.57703	2.061523	0.003247	0.021	523	tags=24%, list=3%, signal=23%	SNAP91/FAM155B/FGF9/KIF5C/DOCK4/ETV1
KRAS.KIDNEY_UP.V1_UP	72	0.603879	2.833815	0.003861	0.0227	579	tags=28%, list=4%, signal=27%	PLP1/PCDH9/SNAP91/GRIA2/SCN2A/CRYAB/FGF9/SOBP/NRN1/MEF2C/RAPGEF4/ANK2/GRIK2/KIF5C/GPM6A/CPEB3/NMNAT2/ETV1/CPE/DDX6
PIGF_UP.V1_DN	114	0.340407	1.710782	0.004566	0.0245	1823	tags=24%, list=11%, signal=21%	FOXG1/DLGAP1/CACNA1C/MSI1/NCAM1/DDX3Y/NPAS3/NPR2/PPFIA4/LOXL1/FZD7/NOS1AP/CREBZF/CEBPA/CDKN2C/EFNA5/SRCAP/SPTB/ANGEL1/BMP2/UBE2H/MAST1/MCF2L/SMARCD1/SMG6/TMEM158/VGF
KRAS.600_UP.V1_UP	137	0.379278	1.971842	0.004926	0.0245	887	tags=14%, list=5%, signal=13%	FOXG1/PCDH9/SNAP91/CTNND2/IGF2/FAM155B/SCN2A/FGF9/ZEB1/REEP2/KIF5C/GALR2/DOCK4/NMNAT2/ETV1/SPRY2/DDX6/NR4A3/LPXN
KRAS.AMP.LUNG_UP.V1_UP	45	0.442193	1.838849	0.006757	0.0326	1776	tags=29%, list=11%, signal=26%	NOVA1/FAM155B/NPAS3/DOCK3/SOX12/EFNA3/RAB30/MCF2L/SLC6A6/HSPA1L/CLSTN3/DYNC1I1/HSF1
KRAS.300_UP.V1_UP	69	0.395214	1.837337	0.007634	0.035	579	tags=13%, list=4%, signal=13%	SNAP91/FAM155B/SCN2A/FGF9/KIF5C/DOCK4/ETV1/SPRY2/DDX6
PTEN_DN.V1_UP	90	0.344074	1.661904	0.008547	0.0383	808	tags=17%, list=5%, signal=16%	TMEM151B/HOXB2/LHX2/H2AFY2/HOXC8/NR2F1/SSBP2/BDNF/TUSC3/KALRN/PAX3/GNAO1/ZBTB10/PRKAR2B/NR4A3
LEF1_UP.V1_UP	134	0.283848	1.464685	0.009901	0.0433	1817	tags=19%, list=11%, signal=17%	FOXG1/WT1/SCN5A/PRPH/FGF9/FGF13/FHL1/HOXB2/FNDC4/ZFPM2/LOXL1/NMNAT2/MAOA/PDZD2/PRKAA2/PLEKHO1/MAP1B/EGR3/FLNC/PHLDA3/WWC2/FN1/KRT13/AUTS2/TMEM158/NR3C1
JAK2_DN.V1_UP	73	0.320531	1.508418	0.027778	0.0916	1911	tags=22%, list=12%, signal=19%	DLGAP1/LBX1/CNKSR2/SLC22A17/ZEB1/ATRNL1/DMD/COL6A3/AKAP6/BMP2/CBX8/ZFHX4/PHF14/KLF13/ACTA2/ZNF821
IL2_UP.V1_DN	104	0.284422	1.401242	0.032407	0.1039	654	tags=12%, list=4%, signal=11%	PBX1/UTY/ISL1/MAGI2/RORB/TEK/H2AFY2/PRKG1/PAK3/HDAC5/HOXA5/FZD2
BRCA1_DN.V1_UP	51	0.343359	1.460856	0.038328	0.1144	1374	tags=22%, list=8%, signal=20%	TLX3/APBB1/MAP2/CDKL5/SP4/SIX1/HOXB7/LHX6/PTPRS/RCN3/OSR2

KRAS.BREAST_UP.V1_DN	65	0.320625	1.465008	0.04059	0.1192	1516	tags=18%, list=9%, signal=17%	PBX1/PPM1E/SOBP/PTENP1/MAML3/HOXB5/COL5A2/ELMO1/DTNB/ALOX12B/NFATC4/FGGY

Table S5. Co-occurrence of Mb21d2 with known PIK pathways regulators in HNSCC based on TCGA sequencing data

A	B	Neither	A Not B	B Not A	Both	Log2 Odds Ratio	p-Value	q-Value	Tendency
MB21D2	PIK3CA	315	26	115	40	2.075	<0.001	<0.001	Co-occurrence
PIK3CA	KRAS	320	124	21	31	1.93	<0.001	<0.001	Co-occurrence
MB21D2	KRAS	391	53	39	13	1.298	0.012	0.044	Co-occurrence
MB21D2	CREB5	407	57	23	9	1.482	0.017	0.051	Co-occurrence
AKT1	KRAS	368	76	47	5	-0.957	0.114	0.19	Mutual exclusivity
MB21D2	PTEN	393	59	37	7	0.334	0.366	0.499	Co-occurrence
PIK3CA	PTEN	310	142	31	13	-0.127	0.473	0.536	Mutual exclusivity

Table S6. List of Primers and antibody dilution used in the study

**Primers**	**Forward**	**Reversed**
qPRC primers for Mb21D2	TCCAAGAATTTACGAAGCACGA	TCATCTAAGTCCAGGTCCACCA
Cloning Primers	GTA GAA TTC GCC ACC ATG AAG ATG GCG GCT CCC ACC	GCG GGA TCC GAA AAA TTT GTC ATC AAT TCT G
Confirmatory Primers	GCC TCA TGC AGG CCT ATG AGG CCT GCA AAG C	CGT TTG CAG GCC TCA TAG GCC TGC ATG AGG C
shRNA	CCTGGACTTAGATGAGCTTAACCGGCCTGGACTTAGATGAGCTTA ACTCGAGTTAAGCTCATCTAAGTCCAGGTTTTTG)	
**Antibody**	**Dilution**	Catalog Number
Mb21D2	1:500 (WB), 1:200(IHC)	(NBP1-92101 WB), HPA044026 (IHC)
PIK3CA	1:500(WB)	ab86714
phospho-PIK3CA	1:300(WB)	D-4: sc-8010
AKT	1:500(WB)	B-1: sc-5298
phospho-AKT	1:200(WB)	(B-5: sc-271966
CREB5	1:500(WB)	A14635
KRAS	1:500(WB)	F234: sc-30
PTEN	1:500(WB)	sc-7974
GAPDH	1:1000(WB)	(ab9485)
Anti-Mouse HRP	1:1000(WB)	ChemiconAP124P
Anti- Rabbit HRP	1:10000(WB)	Biolegend 406401
CD44	1:50(IHC) (WB)	NBP1-47368
KI67	1:50(IHC) (WB)	GTX16667
Twist1	1:500 (WB) 1:200(IHC)	GTX16667
Bmi1	1:1000 (WB) 1:200(IHC)	CST #6964
Snail	1:1000	CST# 3879
**Restriction enzymes**	**Brand**	Cat#
BamHI	New Englands BioLabs	R0136S
EcoRI	New Englands BioLabs	R0101S
DPN1	New Englands BioLabs	R0176S
